# The Cyanobacteria-Dominated Sponge *Dactylospongia elegans* in the South China Sea: Prokaryotic Community and Metagenomic Insights

**DOI:** 10.3389/fmicb.2017.01387

**Published:** 2017-07-25

**Authors:** Zhao-Ming Gao, Guo-Wei Zhou, Hui Huang, Yong Wang

**Affiliations:** ^1^Institute of Deep Sea Science and Engineering, Chinese Academy of Sciences Sanya, China; ^2^Key Laboratory of Tropical Marine Bio-Resources and Ecology, South China Sea Institute of Oceanology, Chinese Academy of Sciences Guangzhou, China

**Keywords:** sponge, symbiont, cyanobacteria, poribacteria, the South China Sea

## Abstract

The South China Sea is a special reservoir of sponges of which prokaryotic communities are less studied. Here, a new record of the sponge *Dactylospongia elegans* is reported near the coast of Jinqing Island in the South China Sea, and its prokaryotic community is comprehensively investigated. Sponge specimens displayed lower microbial diversity compared with surrounding seawater. At the phylum level, prokaryotic communities were consistently dominated by Proteobacteria, followed by Cyanobacteria, Chloroflexi, Acidobacteria, Actinobacteria, Gemmatimonadetes, Thaumarchaeota, and Poribacteria. Operational taxonomic unit (OTU) analysis alternatively showed that the most abundant symbiont was the sponge-specific cyanobacterial species “*Candidatus* Synechococcus spongiarum,” followed by OTUs belonging to the unidentified Chloroflexi and Acidobacteria. Phylogenetic tree based on 16S-23S internal transcribed spacer regions indicated that the dominated cyanobacterial OTU represented a new clade of “*Ca*. Synechococcus spongiarum.” More reliable metagenomic data further revealed that poribacterial symbionts were highly abundant and only secondary to the cyanobacterial symbiont. One draft genome for each of the Cyanobacteria, Chloroflexi and Acidobacteria and three poribacterial genomes were extracted from the metagenomes. Among them, genomes affiliated with the Chloroflexi and Acidobacteria were reported for the first time in sponge symbionts. Eukaryotic-like domains were found in all the binned genomes, indicating their potential symbiotic roles with the sponge host. The high quality of the six recovered genomes of sponge symbionts from the sponge *D. elegans* makes it possible to understand their symbiotic roles and interactions with the sponge host as well as among one another.

## Introduction

Sponges (phylum Porifera) are diverse and widespread metazoans of many marine benthic, fresh-water, quasi-terrestrial, and deep-sea ecosystems. In marine habitats, sponges are considered to play ecologically and biotechnologically important roles. Sponges have been known for several decade to be associated with dense and diverse microbial communities (Vacelet and Donadey, 1977). Motivated by the discovery of pharmacologically important bioactive compounds and a keen interest to elucidate the mechanisms involved in the symbiotic relationship, sponge-associated microbes have been extensively studied during these years (Schmitt et al., 2012).

Among the sponge-associated microbes, the cyanobacteria represent one of the most common groups and are considered to play important roles in photosynthesis, nitrogen fixation, UV protection, and defensive toxin production (Taylor et al., 2007; Webster and Taylor, 2012). Identified cyanobacterial sponge symbionts belong to *Synechocystis, Aphanocapsa, Anabaena, Oscillatoria*, and *Synechococcus* (Taylor et al., 2007). Of them, “*Candidatus* Synechococcus spongiarum,” proposed by Usher et al. (2004), was detected in at least 40 sponge species and constituted the largest sponge-specific cluster to date (Simister et al., 2012). This cyanobacterial symbiont distributed across various geographical regions including the Red Sea, the Mediterranean, the eastern Atlantic, the Caribbean, and the Great Barrier Reef (Simister et al., 2012; Luter et al., 2015). Despite their widespread distribution, 16S rRNA genes of “*Ca*. Synechococcus spongiarum” showed low levels of genetic divergence. However, phylogenetic analysis of the 16S-23S internal transcribed spacer (ITS) regions indicated their high intra-species variations, and 12 distinct clades were found (Erwin and Thacker, 2008). Two more clades were reported in the following genome-level research (Burgsdorf et al., 2015). Other new clades of “*Ca*. Synechococcus spongiarum” should be identified following potential new records of the cyanobacterial sponge symbionts from distinct geographical regions such as the South China Sea.

Due to technical challenges in cultivation of the vast majority of sponge symbionts including “*Ca*. Synechococcus spongiarum,” knowledge of their symbiotic and adaptive mechanisms was limited. Recently, the development of new metagenomic methods however has facilitated the work to recover genomes of uncultured sponge symbionts (Albertsen et al., 2013). The draft genome of “*Ca*. Synechococcus spongiarum” extracted from the Red Sea sponge *Carteriospongia foliascens* provided the first insight into its mutualistic relationship with the sponge host (Gao et al., 2014b). Later work further summarized its general adaptive strategies using three additional draft genomes, one from the Red Sea sponge *Theonella swinhoei* and the other two from the Mediterranean sponge *Ircinia variabilis* Schmidt 1862 and *Aplysina aerophoba* Nardo 1833 (Burgsdorf et al., 2015). Another draft genome of “*Ca*. Synechococcus spongiarum” was also extracted from the South China Sea sponge *Theonella swinhoei* (Liu et al., 2017). Despite impressive improvements regarding knowledge of “*Ca*. Synechococcus spongiarum,” their phylogenomic variations have remained less well explored in comparison to their high intra-species diversity, as indicated by the ITS regions. More genomes of “*Ca*. Synechococcus spongiarum” must be acquired for their phylogenomic and subsequent evolutionary research.

Microbes in sponges are dense and diverse, forming complex mutual relationships. A recent work investigating the deep-see glass sponge *Lophophysema eversa* serves as an ideal example of the interactive network of chemoautotrophic sponge symbionts with respect to nutrient conversions (Tian et al., 2016). For sponges predominantly inhabited by cyanobacteria, other microbes also maintain interactions with the sponge host and play important symbiotic roles. It is valuable to elucidate the whole prokaryotic community of the sponge host and extract genomes of other symbionts to achieve a comprehensive understanding of the symbiotic network associated with cyanobacterial inhabitants, which however has been less concerned to date.

**Figure 1:**
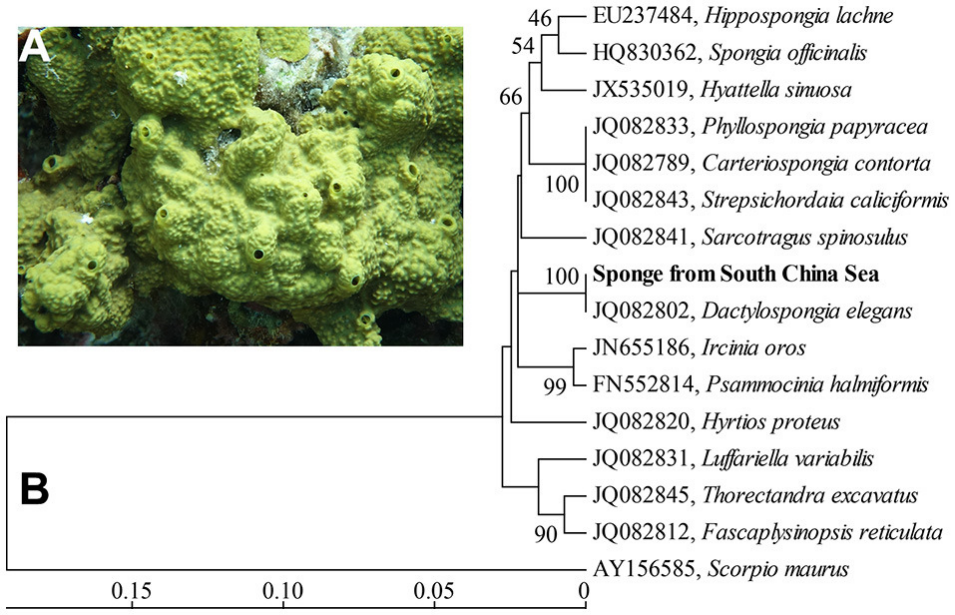
*In situ* photo of the sponge *D. elegans* **(A)** and COXI-based phylogenetic tree **(B)**. The tree was constructed using the neighbor-joining method. Bootstrap values are expressed based on 1,000 replicates. Bar, 5% estimated sequences divergence.

Sponges are widespread in the South China Sea. Reports have described numerous natural products that are extracted from sponges (Karuppiah et al., 2015; Jiao et al., 2016). Sponge-associated microbes have also been reported in several works (Yang and Li, 2012; Sun et al., 2015). However, systematic studies of sponge-associated prokaryotic communities in the South China Sea, especially those associated with cyanobacterial symbionts such as “*Ca*. Synechococcus spongiarum,” remain limited. Here, sponge specimens of *Dactylospongia elegans* were collected from the South China Sea (Figure 1A). Sponge-associated prokaryotic communities were investigated using barcoded 16S rRNA gene amplicons with next generation sequencing methods, which revealed the high abundance of the cyanobacterial symbiont “*Ca*. Synechococcus spongiarum.” More reliable metagenomic method was also used to resolve the prokaryotic community, and highly abundant poribacterial symbionts were revealed. Several nearly complete genomes of sponge symbionts were further retrieved from metagenomes, providing an opportunity to reveal the complex symbiotic network in the sponge *D. elegans*.

## Materials and methods

### Sample collection and DNA extraction

Sponge samples and seawater were collected in June 2014 by SCUBA diving at a depth of ~20 m on the reef flat of Jinqing Island in the South China Sea (16.4424′N, 111.7269′E). The sponge taxonomy was phylogenetically identified using partial subunit I of the cytochrome C oxidase (COXI) amplified according to previously mentioned methods (Erpenbeck et al., 2012) and almost full-length 18/23S rRNA genes assembled from the following metagenomic data. In total, four separated sponge individuals that were disconnected from one another were detached from the seafloor, placed in clean plastic bags and brought to the diving boat. On the deck, sponge samples were rinsed with 0.22-μm-membrane-filter seawater for several times to remove loosely attached microbes and debris. The flushed tissues for each sponge individual were dissected, cut into more than six small pieces with a sterile razor blade, and frozen in 0.8 mL of extraction buffer at −80°C for DNA extraction (Lee et al., 2011). Three randomly selected pieces for each sponge individual were used as replicate samples in the following studies. 3 × 2 L of seawater around the sampling site, used as three control replicates, were filtered directly through dia. 47 mm, 0.22-μm polycarbonate membranes (Millipore, Massachusetts, UK) to capture microbial cells. The membranes were frozen in 0.8 mL of extraction buffer for DNA extraction. Total genomic DNA was extracted using the modified sodium dodecyl sulfate-based method as described previously (Lee et al., 2011). Extracted DNA products were quantified using the Qubit 2.0 Fluorometer (Life Technologies) and stored at −20°C until use.

### Miseq sequencing of 16S rRNA genes

The 16S rRNA gene library for collected sponges and seawater samples was prepared using the 16S Metagenomic Sequencing Library Preparation protocol provided by Illumina, Inc. with several modifications. First, the V3-V4 hypervariable regions of the 16S rRNA genes for each sample were amplified using the forward fusion primer 341F (5′-forward overhang adapter-CCTAYGGGRBGCASCAG-3′) and reverse fusion primer 802R (5′-reverse-overhang adapter-TACNVGGGTATCTAATCC-3′) (Claesson et al., 2010; Klindworth et al., 2013). A 50 μL of PCR reaction mixture consisted of 1.25 U of PrimerSTAR HS DNA Polymerase (Takara, Japan), 1 × PrimerSTAR reaction buffer, 0.2 mM of dNTPs (TaKaRa, Japan), 0.1 μM of primer pairs and 5–10 ng of genomic DNA template. PCR was performed using a thermal cycler (Bio-Rad, USA) with the following conditions: initial denaturation at 98°C for 10 s; 26 cycles of denaturation at 98°C for 10 s, annealing at 50°C for 15 s and extension at 72°C for 30 s; and a final extension at 72°C for 5 min. PCR products were purified using AMPure XP beads (Beckman Coulter, USA). Purified PCR products were subjected to a second-round PCR amplification with the index primers provided with the Nextera XT Index Kit (Illumina, Inc. USA) and using the default PCR conditions provided in the protocol. PCR products were purified using AMPure XP beads in two rounds with a 0.7 ratio, quantified using the Qubit 2.0 Fluorometer (Life Technologies, USA), pooled equally and loaded on an Illumina Miseq (Illumina, Inc. USA) 2 × 300 flow cell at 15 pM using the Miseq reagent kit V3 (Illumina, Inc. USA).

### Prokaryotic community analysis

When the sequencing was finished, Miseq build-in programs automatically separated each sample according to their attached index sequences. Raw paired-end reads were trimmed using Btrim (Kong, 2011) with default parameters and assembled using FLASH with at least 40 bp of overlap (Magoc and Salzberg, 2011). Assembled reads were subjected to QIIME 1.8.0 pipelines for downstream bioinformatics analysis (Caporaso et al., 2010b). First, assembled reads were clustered using UCLUST (Edgar, 2010), and operational taxonomic units (OTUs) were picked at a 97% similarity. Singleton OTUs and those not found more than 10 times in total were removed to minimize potential artifacts (Bokulich et al., 2013). The most abundant reads for the remaining OTUs were selected as representatives and then aligned using PyNAST (Caporaso et al., 2010a) against the SILVA111 database (Pruesse et al., 2007). ChimeraSlayer was used to identify and discard chimeric reads (Haas et al., 2011). Taxonomic assignment was performed using the Ribosomal Database Project (RDP) classifier version 2.2 (Wang et al., 2007) against the SILVA111 database with a threshold value of 0.5. Representatives annotated as chloroplast, mitochondria, and eukaryotes were filtered out. Taxonomic abundance was summarized at the phylum, class, order, family, genus and OTU levels. Species diversity, Shannon index, richness and rarefaction curves were computed using the QIIME alpha diversity pipeline with a step size of 100 and 100 repetitions per step. Relative abundance data for the OTUs were loaded into PRIMER-E, and non-metric multidimensional scaling (nMDS) was performed to compare the prokaryotic community dissimilarity among sponge samples using the Bray-Curtis distance (Clarke and Gorley, 2015). For heatmap clustering, relative abundance data of OTUs were loaded into Cluster3 (de Hoon et al., 2004). OTUs with a relative abundance of less than 0.5% among all samples were filtered out. The remaining OTUs were normalized, centered by the mean, and clustered using the complete linkage method and a metric of correlation (uncentered). A heatmap was then generated with Java TreeView.

### Metagenomic sequencing and assembly

DNA samples of three replicates for each sponge individual were equally pooled and subjected to the Illumina TruSeq Nano DNA Sample Prep Kit to construct a metagenomic library with a 550-bp insert size following the standard protocol. Metagenomic libraries were quantified using the Qubit 2.0 Fluorometer and loaded on the Illumina Miseq (Illumina, Inc. USA) sequencing platform for sequencing. Raw reads were subjected to quality filtering using the IlluQC.pl script in the NGS QC Toolkit under default parameters (Patel and Jain, 2012). Reads smaller than 50 bp and containing more than five ambiguous nucleotides were removed. Qualified reads of each data set were assembled separately and/or together using SPAdes (Bankevich et al., 2012) with a kmer set of 21, 33, 55, 77, and 99 to achieve the best assembly results. Contigs longer than 2,000 bp in each assembled dataset were used for the subsequent genome binning.

### Genome binning

Draft genome binning was carried out mainly based on the genome coverage and tetranucleotide frequency patterns according to a previously described method (Albertsen et al., 2013) but with several modifications. The metagenomic reads of each data set were separately mapped to the assembled contigs using Bowtie2 (Langmead and Salzberg, 2012), and the respective genome coverage was calculated with SAMtools (Li et al., 2009) and manual Perl scripts. The tetranucleotide frequency of the assembled contigs was calculated using Perl scripts written by Albertsen et al. (2013). Principal component analysis of the tetranucleotide frequency was performed using the Vegan package 2.0-5. Open reading frames (ORFs) of the assembled contigs were predicted using Prodigal (Hyatt et al., 2010). A set of 107 Hidden Markov Models (HMM) of conserved proteins (Albertsen et al., 2013) was searched against the predicted ORFs with default cutoff values in the HMM datasets. The identified conserved proteins were searched against the NCBI NR database using the BLASTP program (*e*-value of 1e-05) and taxonomically assigned using MEGAN 5.0 (Huson et al., 2011). Contigs were labeled according to the phylum-level taxonomic affiliation of the conserved proteins. Relying on these data, draft genomes of sponge symbionts were extracted from the assembled contigs using RStudio with the previously described R pipelines (Albertsen et al., 2013). Number of conserved proteins detected was used to evaluate the completeness of the binned genomes.

### Genome analysis

For the KEGG annotation, the predicted amino acid sequences of the extracted draft genomes and other reference genomes downloaded from the NCBI Genome database were searched against the KEGG database (Kanehisa et al., 2012) using BLASTP with a maximum *e*-value cutoff of 1e-05. Amino acid sequences were also searched against the GenBank NR database, and the output xml file was imported into MEGAN for taxonomic affiliation and SEED/Subsystems annotation (Overbeek et al., 2005). KEGG and SEED/Subsystems annotations of the sponge symbionts and close relatives were compared to evaluate the genome reduction. Eukaryotic-like domains, including ankyrin repeats (ARs), tetratricopeptide repeats (TPRs), leucine rich repeats (LRRs), NHL repeats, fibronectin type III, and cadherins, were annotated using pfam_scan.pl script by searching against the PFAM database (Punta et al., 2012) according to a previously described method (Fan et al., 2012).

### Phylogenetic tree construction

For phylogenetic analysis, the targeted gene sequence was searched against the NCBI GenBank database using BLASTN to detect closely related relatives. A neighbor-joining tree was constructed using MEGA5.1 software (Tamura et al., 2011). Multiple alignment was performed using ClustalW (Thompson et al., 1994). Distance matrices were calculated using Kimura's two-parameter correction model (Kimura, 1980). Bootstrap values were determined with 1,000 replications.

### Accession numbers of nucleotide sequences

The COXI and 18S/23S rRNA gene sequences of the sponge *D. elegans* are stored in the NCBI GenBank database under accession numbers KY979509, KY970157, and KY970158, respectively. Raw data for the 16S rRNA gene library and metagenomes are available in the NCBI Sequence Reads Archive (SRA) database under BioProject accession number PRJNA383957.

## Results

### Sponge identification

A phylogenetic tree based on partial COXI gene sequences (579 bp) showed a high level of conservation (100% identity) between the sponge in this study and the sponge *Dactylospongia elegans* (Figure 1B). Further phylogenetic analysis using 18/23S rRNA gene sequences also indicated that the sponge in this study was closely related to the sponge *D. elegans* (Supplementary Figures 1, 2). These data supported the classification of the sponge from the South China Sea as a new record of the sponge species *D. elegans*.

### Prokaryotic community analysis

#### Prokaryotic richness and diversity

In total, 16S rRNA amplicons of fifteen samples, including three seawater samples and twelve sponge specimens that consisted of three replicates for each of the four sponge individuals, were subjected to Illumina sequencing. The final datasets and the number of OTUs, Chao1 estimation of species richness and Shannon index are summarized (Table 1). After quality control, 1,398,381 assembled reads were obtained, and the smallest dataset is 62,322 reads referred to the sponge sample SP1-2. Rarefaction curves, drawn based on OTUs at a 3% dissimilarity, showed that all the samples represented the prokaryotic communities very well (Supplementary Figure 3). In comparison to the seawater samples with 922–969 OTUs, the sponge specimens displayed a lower microbial diversity with 366–580 OTUs per sample. The divergences of prokaryotic community were further confirmed using a normalized data size of 61,489 reads that covered the samples with the lowest sequencing depth (Table 1). The normalized number of both OTUs and Chao1 showed remarkably lower microbial diversity in sponge samples than in seawater samples. However, the Shannon index did not clearly differ between sponge specimens and seawater samples. On the other hand, the microbial diversity among sponge individuals was nearly consistent. An exception was sponge individual #3, which showed a higher Chao1 than individual #2 and #4 and a larger number of OTUs compared with individual #2.

**Table 1:** Diversity summary of microbial communities in the sponge and seawater.

**Sample ID**		**Number of qualified reads**	**Total reads**			**Normalized 61489 reads**		
			**OTUs**	**Chao1**	**Shannon**	**OTUs**	**Chao1**	**Shannon**
#1	SP1-1	82,645	540	681	5.0	493	661	5.0
	SP1-2	62,323	372	520	4.4	370	520	4.4
	SP1-3	83,019	454	548	4.8	416	531	4.8
#2	SP2-1	102,703	385	481	4.8	329	449	4.8
	SP2-2	89,121	366	474	5.0	322	457	5.0
	SP2-3	67,517	420	560	5.3	407	550	5.3
#3	SP3-1	97,500	504	632	4.8	432	605	4.8
	SP3-2	115,863	580	653	4.7	483	627	4.7
	SP3-3	93,102	523	624	4.9	465	588	4.9
#4	SP4-1	81,331	464	547	5.1	429	536	5.1
	SP4-2	112,489	385	479	4.9	313	454	4.9
	SP4-3	84,738	392	519	4.8	351	498	4.8
Sea water	SW-1	124,033	922	946	4.7	844	930	4.7
	SW-2	81,075	969	1,002	5.5	946	992	5.5
	SW-3	120,922	963	978	5.2	908	964	5.2

*OTUs, Shannon index and Chao1 were determined at a 3% dissimilarity. SP1, SP2, SP3, and SP4 are referred to four individuals of the sponge D. elegans. “−1,” “−2,” and “−3” denote three technical replicates, respectively*.

#### Taxonomic abundance at the phylum level

**Figure 2:**
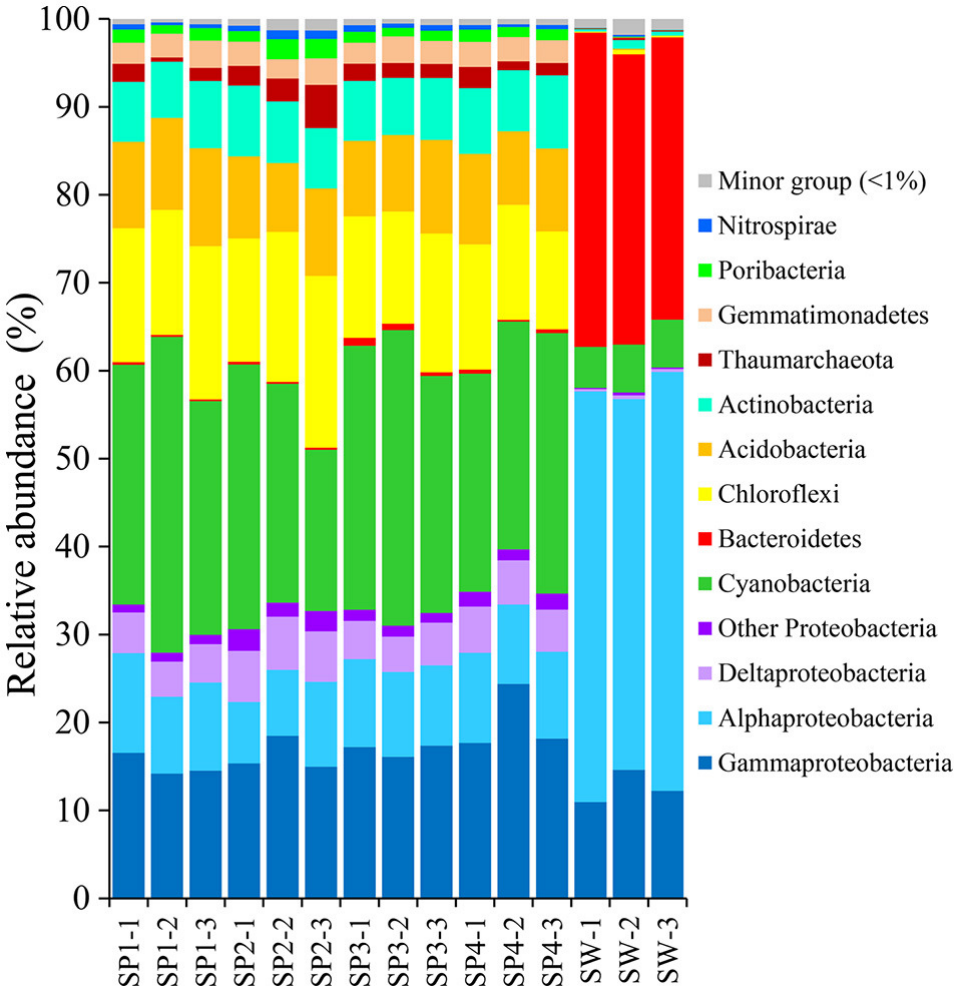
Taxonomic abundance of microbial reads in sponges and seawater at the phylum level. Microbial reads of 16S rRNA gene amplicons were assigned taxonomically using the RDP classifier against the SILVA 111 database with a confidence threshold of 50%. Sample IDs are referred to Table 1.

With the QIIME pipelines, the taxonomic abundance of Illumina assembled reads was summarized at the phylum level (Figure 2 and Supplementary Table 1). In total, 17 prokaryotic phyla were consistently detected in almost all the sponge samples, and the dominant phyla were Proteobacteria (32.81 ± 2.95%, mainly composed of Gammaproteobacteria with a composition of 17.10 ± 2.71%, Alphaproteobacteria with a composition of 9.35 ± 1.19% and Deltaproteobacteria with a composition of 4.90 ± 0.69%), followed by Cyanobacteria (27.82 ± 4.53%), Chloroflexi (14.85 ± 2.30%), Acidobacteria (9.55 ± 1.02%), Actinobacteria (7.15 ± 0.60%), Gemmatimonadetes (2.70 ± 0.28%), Thaumarchaeota (1.99 ± 1.10%, Archaea), and the candidatus phylum Poribacteria (1.41 ± 0.43%), resulting in a total of more than 98% of all qualified sequences. Nitrospirae was also detectable in all the sponge samples, with a relative abundance of 0.60 ± 0.17%. Given slight dynamics of the relative abundance, there were no obvious divergences among sponge individuals in the composition of the dominant phyla. Alternatively, the dominant phyla in seawater samples belonged to Proteobacteria (Gammaproteobacteria and Alphaproteobacteria), Bacteroidetes and Cyanobacteria, demonstrating a simplex phylum-level composition (Figure 2).

#### Highly abundant OTUs and phylogenetic relationships

**Figure 3:**
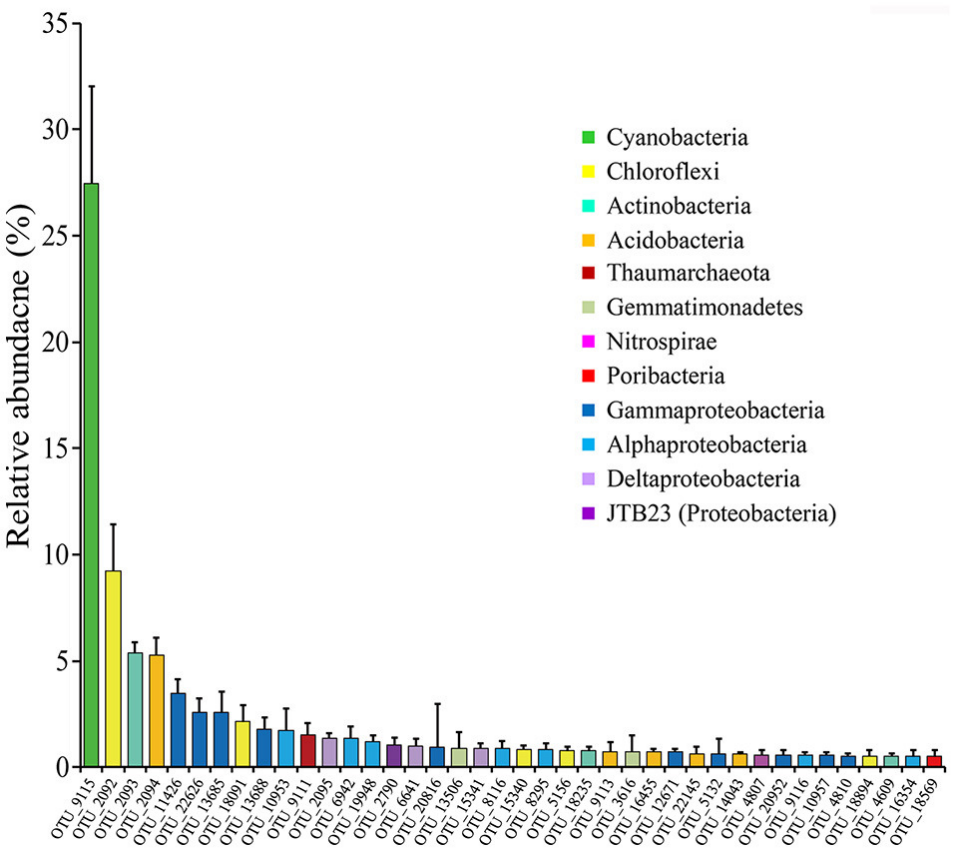
The relative abundance of OTUs with average proportions of more than 0.5%. The relative abundance of OTUs in twelve sponge specimens of four sponge individuals were averaged and the Standard Deviations (SD) were shown with error bars.

**Figure 4:**
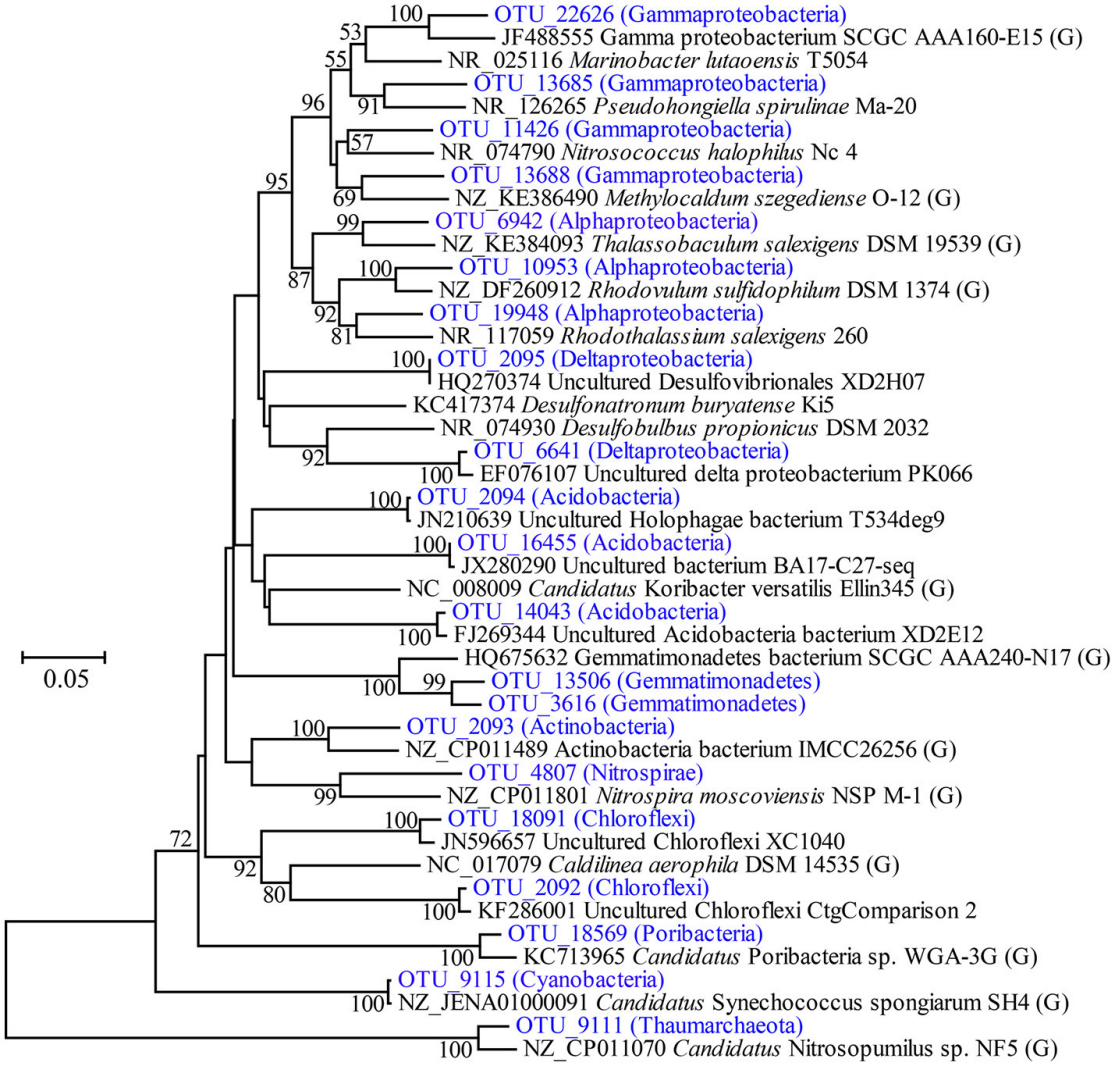
Phylogenetic tree of representative highly abundant OTUs and their relatives. The tree was constructed based on partial 16S rRNA gene sequences by the neighbor-joining method. Bootstrap values are expressed based on 1,000 replications, and only values more than 50% are shown. Bar, 5% estimated sequences divergence. The abundance of OTUs included on the tree are shown in Figure 3. The brackets including “G” indicates strains with reported genomes.

Highly abundant OTUs with average proportions of more than 0.5% among sponge specimens are summarized (Figure 3 and Supplementary Table 2). The most abundant OTU was OTU_9115 in the phylum Cyanobacteria, which accounted for proportions of 27.45 ± 4.55% in the sponge-associated prokaryotic communities and shared the highest similarity (>99%) to the sponge-specific cyanobacterial symbiont “*Ca*. Synechococcus spongiarum” (Figure 4). Phylogenetic analysis of the 16S-23S ITS regions further indicated that OTU_9115 represented a new clade of “*Ca*. Synechococcus spongiarum” (Supplementary Figure 4). Following the cyanobacterial symbiont in terms of relative abundance were OTU_2092 and OTU_18091 in the phylum Chloroflexi, OTU_2093 in Actinobacteria, and OTU_2094 in Acidobacteria. The phylum Proteobacteria was divided into more than 20 OTUs mainly belonging to Gammaproteobacteria, Alphaproteobacteria and Deltaproteobacteria, and lost the dominated position. Abundant OTUs also included OTU_9111 in the phylum Thaumarchaeota, OTU_13506 and OTU_3616 in Gemmatimonadetes, OTU_4807 in Nitrospirae, and OTU_18569 in Poribacteria. Among them, OTU_9111 was most closely related to an ammonia-oxidizing archaea “*Ca*. Nitrosopumilus sp. NF5” with an identity of 96.06%. OTU_18569 was affiliated with “*Ca*. Poribacteria sp. WGA-3G” with an identity of 96.72%. Two Gemmatimonadetes OTUs shared 96% identity with each other and 93% identity with the closest relative Gemmatimonadetes bacterium SCGC AAA240-N17. Other highly abundant OTUs were distantly related to known taxa with an identity of less than 93%, a threshold that is usually used to define genera (Figure 4 and Supplementary Figure 5). In particular, all five OTUs in the phylum Chloroflexi shared an identity of less than 88% with known taxa (Figure 4 and Supplementary Figure 5). Seawater samples were alternatively enriched for very distinct OTUs (Supplementary Table 2).

#### OTU-level diversity of sponge associated prokaryotic communities

**Figure 5:**
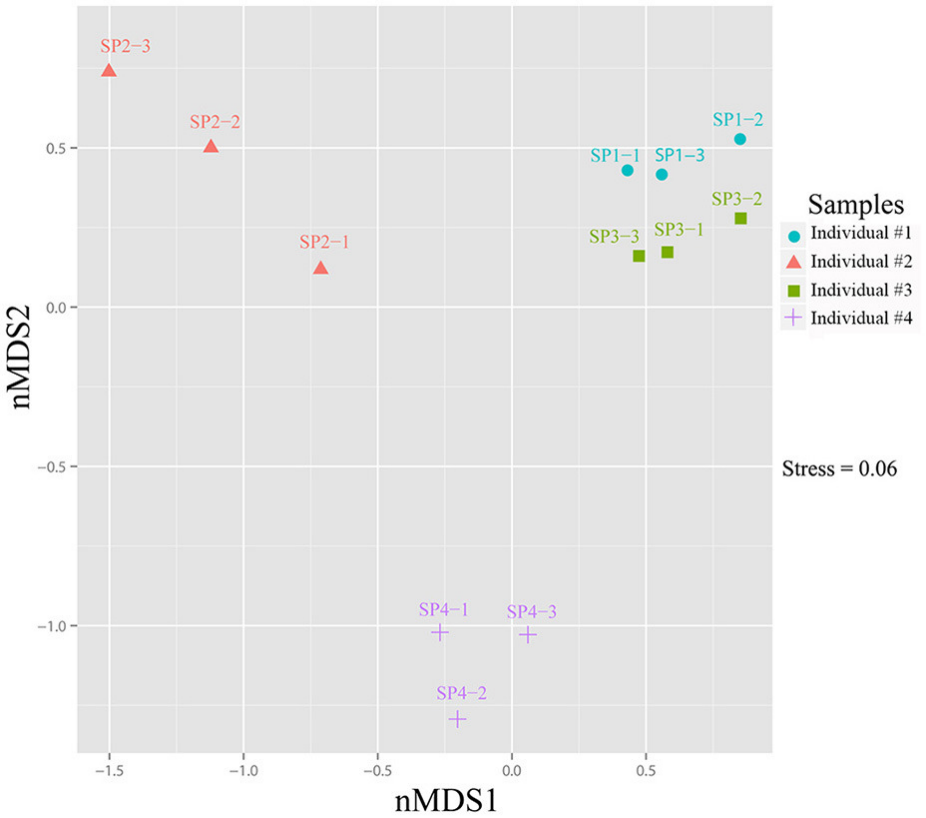
Non-metric multidimensional scaling (nMDS) ordination of sponge-associated prokaryotic communities. The two-dimensional stress value for the nMDS was 0.06 based on the Bray-Curtis distance. Analysis was performed using PRIMER-E based on OTU-based relative abundance. Plots were produced with ggplot2 (Wickham, 2009) in the R environment (http://www.R-project.org).

Heatmap clustering revealed that the relative abundance of OTUs was highly consistent among the three replicates of each sponge individual but varied to a certain degree among sponge individuals (Supplementary Figure 6). Although the top six abundant OTUs, including the cyanobacterial the OTU_9115, showed no significant differences among sponge individuals (*T*-test: *p* > 0.05), the relative abundance of a large number of the following OTUs varied variable among sponge individuals (Supplementary Table 2). Based on the divergence in OTU abundance, sponge individuals were separated into two clusters, one composed of individuals #1 and #3, and another composed of #2 and #4 (Supplementary Figure 6). The non-metric multidimensional scaling (nMDS) plot further clearly discriminated the prokaryotic communities among sponge individuals into three groups with a stress value of 0.06 (Figure 5).

#### OTUs in the phylum poribacteria

**Figure 6:**
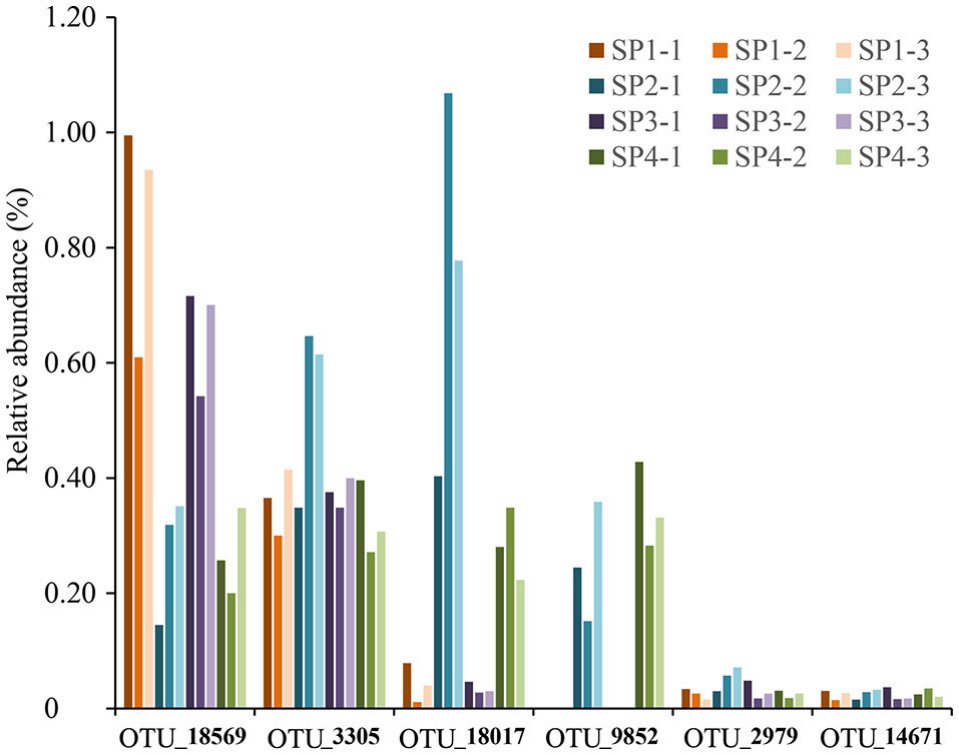
The relative abundance of OTUs affiliated with the phylum Poribacteria. All six poribacterial OTUs in the sponge *D. elegans* were included. Sample IDs are referred to Table 1.

In total, six poribacterial OTUs were recovered from sponge specimens (Figure 6). Their proportions showed variable patterns among sponge individuals. OTU_18569 and OTU_3305 were relatively highly abundant in all sponge individuals. The abundance of OTU_18569 was especially higher in individuals #1 and #3 compared with individuals #2 and #4. OTU_18017 and OTU_9852 only showed a high abundance in individuals #2 and #4. OTU_2979 and OTU_14671 were less abundant in all sponge samples. Phylogenetic tree of partial 16S rRNA gene sequences confirmed the affiliation of the six OTUs with the phylum Poribacteria, three of which were closely related to reported species that owned draft genomes (Kamke et al., 2013). The other three OTUs, including OTU_18569, OTU_3305 and OTU_14671, however, fell into separated novel clades (Supplementary Figure 7).

### Metagenomic analysis

#### Genome binning

**Figure 7:**
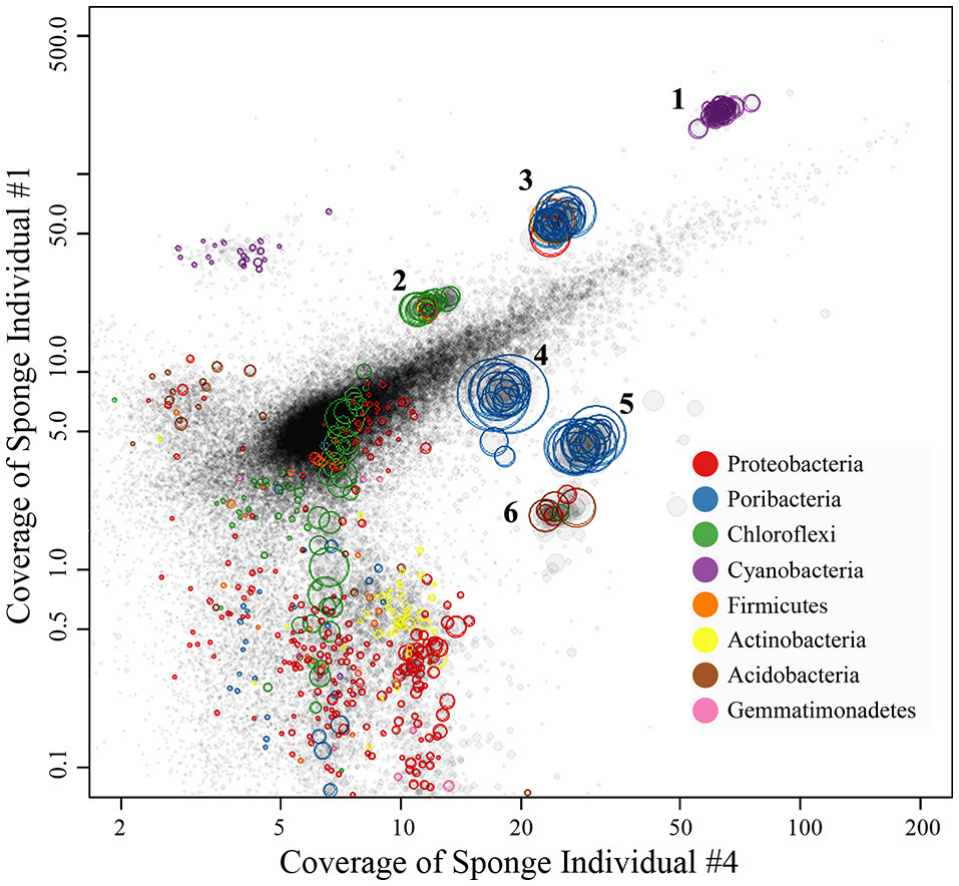
Binned genomes of highly abundant symbionts in the sponge *D. elegans*. Combined assembly of qualified reads of sponge individual #2 (META2) and individual #4 (META4) produced the contigs, which were mapped to the X-axis with their converage in META4 and to the Y-axis with their converage in individual #1 (META1). Each circle represents a contig with the area indicating the length and the color indicating its taxonomy. Contigs were grouped into draft genomes based on the homogeneous coverage and the GC content. The draft genomes were further refined by examining consistency in tetranucleotide frequency of contigs.

Four metagenomic datasets, META1, META2, META3, and META4, corresponding to four sponge individuals, were obtained using Illumina-based high throughput sequencing. Datasets were assembled separately and/or together for the best bins of target symbionts. Assembly of META2 produced the best draft genome for the cyanobacterial symbiont, which was 2.15 Mbp in length with a GC content of 61.9% and composed of a total of 51 contigs (Table 2). 16S rRNA gene sequence derived from the bin was homologous to OTU_9115, and thus this cyanobacterial symbiont was named “*Ca*. Synechococcus spongiarum” strain DE9115. Assembly of META2 and META4 produced the best draft genomes for the other five highly abundant sponge symbionts belonging to phyla Chloroflexi, Acidobacteria and Poribacteria (Table 2 and Figure 7). 16S rRNA gene sequences of the Chloroflexi and Acidobacteria bins were homologous to OTU_2092 and OTU_2094, the most abundant OTUs of the respective phyla in the sponge. Both displayed a large distance from known relatives (Figure 4), and no lower-level taxonomy could be assigned. Thus, the draft genomes were named Chloroflexi sp. DE2092 and Acidobacteria sp. DE2094, respectively. The other three were poribacterial genomes (named POR1, POR2, and POR3). No valid 16S rRNA gene sequence were retrieved from the poribacterial genomes.

**Table 2:** Summarized characteristic of binned genomes of the sponge symbionts.

**Genome features**	**Values for indicated taxa**^a^					
	**1**	**2**	**3**	**4**	**5**	**6**
Genome size (Mbp)	2.15	3.81	5.16	5.11	5.96	4.2
No. of contigs	51	110	48	29	40	69
No. of conserved genes^b^	105/104	104/102	108/103	104/102	110/104	105/101
Genome recovery	97.2%	95.3%	96.3%	95.3%	97.2%	94.4%
% GC content	61.9	59.3	47.9	41.9	47.4	68.7
% Coding density	90.6	89.8	89.5	90.0	88.5	94.4
**EUKARYOTIC-LIKE DOMAIN**						
Fibronectin type III	3	34	2	5	2	44
Cadherin	0	0	13	17	6	0
AR	1	2	17	8	17	4
TPR	6	25	141	187	145	76
LRR	25	0	114	278	110	2
NHL	0	1	12	6	10	0
**RELATIVE ABUNDANCE**^c^						
Individual #1	1	0.11	0.28	0.04	0.02	0.01
Individual #2	1	0.15	0.22	0.05	0.15	0.02
Individual #3	1	0.05	0.21	0.10	0.08	0.19
Individual #4	1	0.19	0.39	0.28	0.46	0.39
Respective OTUs	9115	2,092	/	/	/	2,094

a *Taxa: 1, “Ca. Synechococcus spongiarum” DE9115; 2, Chloroflexi sp. DE2092; 3, Poribacteria sp. POR1; 4, Poribacteria sp. POR2; 5, Poribacteria sp. POR3; 6, Acidobacteria sp. DE2094*.

b *Number: Total/unique conserved genes*.

c *Relative abundance: Using the coverage of the cyanobacterial genome as a reference, ratios of the coverage of the other binned genomes to the reference were used to indicate their relative abundance in respective samples*.

#### Genome completeness and contamination

According to the number of 107 single-copy conserved genes present in sponge symbionts (Albertsen et al., 2013), all six binned genomes were assumed to be recovered for more than 94% and up to 97.2% (Table 2). One duplicate conserved gene was found in the cyanobacterial genome DE9115 (TIGR00436), two duplicate conserved genes in Chloroflexi sp. DE2092 (TIGR00442, TIGR00436), and four duplicate conserved genes in Acidobacteria sp. DE2094 (PF00347.18, TIGR00436, PF01795, and PF00750). TIGR00436, PF01795, and PF00750 were not consistently present as a single copy (Albertsen et al., 2013). Thus, potential contaminations in the above three genomes should be low. Two duplicate conserved genes were observed in poribacterial genome POR2, and up to six in both poribacterial genomes POR1 and POR3. However, the ever reported poribacterial genome Poribacteria sp. 3G also had a large number of duplicated conserved genes that were assumed to be present as a single copy (Kamke et al., 2013), indicating a genome characteristic of this phylum but not contamination.

#### Genome-level relative abundance

Relative abundance of binned genomes among sponge individuals was normalized using the cyanobacterial symbiont DE9115 as a reference and is shown in Table 2. DE9115 was consistently predominant among four metagenomes. The following abundant symbiont was poribacterial POR1, which also showed a high relative abundance in all four metagenomes and was only secondary to cyanobacterial DE9115 in three of them. Alternatively, poribacterial POR3 was the secondary abundant genome in the fourth metagenome. Poribacterial POR2 was less abundant than POR1 and POR3. Chloroflexi sp. DE2092 was the third abundant genome in two metagenomes and Acidobacteria sp. DE2094 was the third in another two metagenomes. In summary, Poribacteria could be considered as another predominant symbiont in the sponge *D. elegans* following cyanobacterial DE9115 due to their consistently high abundance in the four metagenomes.

#### Eukaryote-like domains

The abundance of six eukaryotic-like domains in binned genomes was analyzed (Table 2). Fibronectin type III domains, ARs and TPRs can be found in all the binned genomes. LRRs were missing in Chloroflexi sp. DE2092. NHLs were missing in cyanobacterial DE9115 and Acidobacteria sp. DE2094. Cadherins were only found in poribacterial genomes. On the other hand, Chloroflexi sp. DE2092 and Acidobacteria sp. DE2094 had a greater number of Fibronectin type III domains. Alternatively, poribacterial genomes had a larger number of the other eukaryotic-like domains, as highlighted by up to 187 TPRs and 278 LRRs in POR2. Since Chloroflexi sp. DE2092 shared a far distant similarity with *Caldilinea aerophila* (Figure 4), the number of eukaryotic-like domains in DE2092 and typical Chloroflexi relatives was summarized (Supplementary Table 3). Ankyrin repeats were only found in DE2092, and the number of Fibronectin type III domains was obviously higher in DE2092 than in close relatives. Unfortunately, there were no sufficient close relatives that could be used to compare the number of eukaryotic-like domains in the Acidobacteria symbiont DE2094.

## Discussion

The sponge *D. elegans* (Thiele, 1899)^1^ is reported to be distributed in Banda Sea, Malacca Strait, Palau, Sulawesi Sea/Makassar Strait, West Caroline Islands, Indonesia and Singapore, according to the records of the *World Porifera Database*, as well as in the Okinawa Ocean (Mitome et al., 2001). Here, a new record was reported in the South China Sea. New natural products such as sesquiterpene benzoxazoles and sesquiterpene quinones have been isolated from this sponge species (Ovenden et al., 2011), whereas the sponge-associated prokaryotic communities have not been studied to date. The present study provides the first comprehensive investigation of the prokaryotic community of the sponge *D. elegans*.

Based on 16S rRNA gene sequencing, specimens of sponge *D. elegans* displayed consistently lower microbial diversity compared with the surrounding seawater, indicating simplex prokaryotic community compositions and a characteristic belonging to the Low Microbial Abundance sponges (Giles et al., 2013). The prokaryotic communities of four sponge individuals showed no obvious discrepancies at the phylum level and were consistently predominated by Proteobacteria. However, the relative abundance of OTUs was divergent among sponge individuals, and nMDS analysis further discriminated sponge-associated prokaryotic communities into three groups (Figure 5 and Supplementary Figure 6), which indicated intra-species variations of prokaryotic communities and probably owed to their different microenvironments (Gao et al., 2014a; Hardoim and Costa, 2014). On the other hand, the cyanobacterial symbiont “*Ca*. Synechococcus spongiarum” was always the most abundant OTU, suggesting a cyanobacteria-dominated prokaryotic community. An uncultured bacterium in the phylum Chloroflexi (OTU_2092) was ranked second in relative abundance. Alternatively, metagenomic data that were more reliable than the 16S rRNA-based method revealed the high abundance of bacteria in the phylum Poribacteria and only secondary to “*Ca*. Synechococcus spongiarum.”

As one of the biggest sponge-specific clusters, “*Ca*. Synechococcus spongiarum” has been found in at least 40 sponge species and formed more than 12 clades based on the divergence of the ITS regions (Erwin and Thacker, 2008; Simister et al., 2012; Burgsdorf et al., 2015). Five draft genomes have been obtained, including one from the South China Sea (Burgsdorf et al., 2015; Liu et al., 2017). The present study provides another report about this cyanobacterial sponge symbiont (DE9115) in the South China Sea and expands its geographical distribution. Due to geographical separations and sponge host difference, the cyanobacterial symbionts DE9115 in the sponge D. elegans from the South China Sea may evolve independently from others. The phylogenetic tree based on ITS regions indicated that DE9115 form a new clade of “*Ca*. Synechococcus spongiarum” and validated its potential distinction. The high relative abundance of this cyanobacterial symbiont further indicated the important roles of photoautotrophy in the sponge *D. elegans*. The draft genome of “*Ca*. Synechococcus spongiarum” DE9115 has been successfully extracted, which represented the best assembled genome reported to date with up to 97.2% completeness and consisting of only 51 contigs (Gao et al., 2014b; Burgsdorf et al., 2015). The new binned genome may be an excellent supplement to illustrate the phylogenomic variations and adaptive evolution of symbiotic cyanobacteria through comparative genomic analysis.

The candidate phylum Poribacteria was originally discovered and described in marine sponges (Fieseler et al., 2004). It has been thus far identified in more than a dozen sponge species and represented another widespread distributed sponge symbionts (Lafi et al., 2009). Six poribacterial OTUs were also found in the sponge *D. elegans* from the South China Sea but showed a very low abundance in all sponge specimens. Interestingly, three poribacterial genomes were extracted from the metagenomic data, which contrarily showed high relative abundance. Poribacteria sp. POR1 was especially highly abundant and only secondary to cyanobacterial symbiont DE9115 in three metagenomes. POR3 was the second most abundant genome in the fourth metagenomes. The low abundance of poribacterial OTUs in prokaryotic communities should be ascribed to the bias of selected primer pairs which led to inefficient amplification of poribacterial 16S rRNA genes. Six poribacterial genomes have been obtained from the Mediterranean sponge *Aplysina aerophoba* using single-cell methods, and common features indicated their heterotrophic lifestyles and potential roles as efficient scavengers and recyclers of the sponge extracellular matrix (Kamke et al., 2013). High abundant poribacterial symbionts in the sponge *D. elegans* may also be considered as recyclers of host extracellular matrix. In terms of the predominant cyanobacterial symbionts and their autotrophic lifestyles via the absorption of light energy, poribacterial symbionts may also play important roles in rearranging the external morphology of sponge host to facilitate the photosynthesis of cyanobacterial symbionts. Only one of the ever obtained single-cell poribacterial genomes was sufficiently high quality with an estimated genomes recovery of 98.5%, but others were fragmented and incomplete (Kamke et al., 2013). Interestingly, all three poribacterial genomes from the sponge *D. elegans* were high quality with estimated completeness of higher than 95%, which will facilitating our understanding of the lifestyle variations and phylogenomic associations of poribacterial sponge symbionts.

Chloroflexi, with five highly abundant OTUs, was another highly abundant phylum inhabiting the sponge *D. elegans*. Chloroflexi formally comprises filamentous anoxygenic photoautotrophs, aerobic organotrophs, thermophilic chemoheterotrophs, and other heterotrophic organisms, representing a physiologically diverse and ubiquitous group of organisms found in a wide range of aquatic and terrestrial environments (Campbell et al., 2014). Chloroflexi has also been frequently reported in sponges and formed numerous sponge-specific and sponge-coral-specific clusters (Schmitt et al., 2011). Despite their dense diversity and specificity in sponges, no genomes of Chloroflexi symbionts have been elucidated. Fortunately, a high-quality draft genome affiliated with OTU_2092, the most abundant Chloroflexi OTU, has been successfully extracted from the sponge *D. elegans*. Since OTU_2092 is phylogenetically distant from known Chloroflexi species, the extracted genome should be helpful to illustrate its symbiotic roles. Preliminary analysis revealed that the Chloroflexi genome has numerous genes associated with carbohydrate transport and metabolism similar to poribacterial symbionts, but no genes associated with carbon fixation were found, which supported its heterotrophic lifestyle. There is a possibility that the Chloroflexi symbiont is a complementary scavenger and recycler of carbon compounds originating from seawater and the sponge host in addition to Poribacteria (Kamke et al., 2013).

Besides the above sponge symbionts, Acidobacteria was also highly abundant in the sponge *D. elegans*, and an almost complete Acidobacteria genome was obtained. The phylum Acidobacteria has also been frequently detected in sponges (Schmitt et al., 2012). Like most of the sponge symbiotic groups, no genome of the Acidobacteria symbionts has been reported until now. The present report gives the first draft genome of sponge symbionts in the phylum Acidobacteria. Since most of the sponge symbionts are uncultivated, it is very valuable to obtain their genomes using alternative metagenomic methods for understanding their symbiotic roles (Webster and Taylor, 2012; Albertsen et al., 2013).

At the phylum level, Proteobacteria was the most dominant taxon of prokaryotic communities in the sponge *D. elegans*. Proteobacteria has been reported to be one of the most diverse phyla of sponge-associated microbial communities (Schmitt et al., 2012), and an Gammaproteobacteria symbiont from the deep-sea glass sponge *Lophophysema eversa* has been confirmed to play important roles in sulfur oxidation pathway (Tian et al., 2016). It is reasonable to think that the predominant Proteobacteria play important symbiotic roles in the sponge *D. elegans*. However, further analysis showed that Proteobacteria was divided into a larger number of OTUs and lost the predominant position. Proteobacteria OTUs were mainly affiliated with Gammaproteobacteria, Alphaproteobacteria, Deltaproteobacteria and JTB clades, but were far distant from known taxa in the phylogenetic tree (Figure 4). Assembled contigs from metagenomic data belonging to Proteobacteria were less abundant in comparison to the successfully binned genomes and were excessively fragmented (the red circles in Figure 7). Preliminary analysis of conserved genes indicated that the contigs mainly belonged to Gammaproteobacteria, but also far distant to known genomes. Further work is needed for improving the assembly quality and obtaining ideal draft genomes in order to illustrate their roles in the sponge.

A Thaumarchaeota OTU was found in the sponge-associated prokaryotic communities and was closely related to ammonia-oxidizing *Nitrosopumilus*. The ammonia oxidizer *Ceratodictyon spongiosum* has been demonstrated to remove the ammonia excreted by the sponge host in the sponge *Haliclona cymaeformis* (Davy et al., 2002). Furthermore, *Nitrosopumilus*-like archaea were predominant in the deep-sea glass sponge *L. eversa* and cold-seep sponge *Suberites* sp., both of which have been proposed to play important roles in ammonia oxidization (Tian et al., 2016, 2017). Similar roles should also be suggested for the Thaumarchaeota in the sponge *D. elegans*. A potential nitrite-oxidizing Nitrospirae OTU was also found and probably further oxidized nitrite to nitrate (Ehrich et al., 1995). However, the abundance of Thaumarchaeota and Nitrospirae was quite lower in comparison to the dominated cyanobacterial and poribacterial symbionts, indicating that their roles may be not so important in the sponge *D. elegans*.

Eukaryotic-like domains, such as fibronectin type III, ARs, TPRs, LRRs, cadherin, and NHL repeats, have been reported to be enriched in sponge symbiotic microbes (Liu et al., 2011; Siegl et al., 2011) and be supposed to modulate host behavior by interfering with eukaryotic protein-protein interactions. Specially, experiments have shown that ARs from sponge symbionts modulated amoebal phagocytosis and were supposed to help symbionts escape digestion by the sponge host (Nguyen et al., 2013). ARs were found in all the six binned genomes of the sponge *D. elegans*. Cyanobacterial and poribacterial symbionts has been reported to contain larger number of ARs (Gao et al., 2014b; Kamke et al., 2014). The present study also showed that ARs were only found in Chloroflexi sp. DE2092 but not its close relatives. These enriched ARs indicated potential intimate relationships of symbionts with the sponge host and may perform similar functions of avoiding digestion by the host. The highest frequency of TPR and LRR domains in poribacterial genomes was consistent with the previous work (Kamke et al., 2014). The number of Fibronectin type III domains was also clearly higher in Chloroflexi DE2092 compared with its close relatives. TPRs, LRRs, and Fibronectin type III have all been reported in pathogens and may play roles in host-pathogen interactions (Fan et al., 2012; Kamke et al., 2014). There is a possibility that these domains also mediated host-symbiont interactions in the sponge *D. elegans*. Enriched eukaryotic-like domains were probably the main factors facilitating the symbionts to inhabit the sponge host.

## Conclusions

Here, a new record of the sponge *D. elegans* was reported in the South China Sea, and its cyanobacteria-predominated prokaryotic community was comprehensively revealed through 16S rRNA gene sequencing. Metagenomes further helped to illustrate the composition of the sponge-associated prokaryotic community, and Poribacteria were found to be the secondary abundant groups after the cyanobacterial symbiont. Meanwhile, excellent genomes of six highly abundant symbionts belonging to Cyanobacteria, Poribacteria, Chloroflexi, and Acidobacteria were extracted from metagenomes, making the sponge a perfect model to understand the complex interactions of sponge-associated symbionts. Eukaryotic-like proteins have been found in binned genomes, and further studies would be helpful to illustrate their potential interactions in the sponge host.

## Author contributions

ZG, HH, and YW designed the research. ZG and GZ performed the experiments. ZG, GZ, and YW analyzed the data. ZG wrote the manuscript with contributions from all authors.

### Conflict of interest statement

The authors declare that the research was conducted in the absence of any commercial or financial relationships that could be construed as a potential conflict of interest.

## Footnotes

^1^http://www.marinespecies.org/porifera/porifera.php?p=taxdetails&id=165312
