# Supplementary material for: The Cyanobacteria-Dominated Sponge Dactylospongia elegans in the South China Sea: Prokaryotic Community and Metagenomic Insights
Source: Front Microbiol. 2017 Jul 25;8:1387. doi: 10.3389/fmicb.2017.01387 (PMC5524777; doi:10.3389/fmicb.2017.01387)
Supplement: Supplementary file 1 [file DataSheet1.DOCX]

**The cyanobacteria-dominated sponge *Dactylospongia elegans* in the South China Sea: prokaryotic community and metagenomic insights**

Zhao-Ming Gao^1^, Guo-Wei Zhou^2^, Hui Huang^2^, Yong Wang^1^

*^1^. Institute of Deep Sea Science and Engineering, Chinese Academy of Sciences, Sanya, Hai Nan, P.R. of China*

*^2^. South China Sea Institute of Oceanology, Chinese Academy of Sciences, Guangzhou, P.R. of China*

**Corresponding author:**

Yong Wang, PhD

Institute of Deep Sea Science and Engineering, Chinese Academy of Sciences

No. 28, Luhuitou Road, Sanya, Hai Nan, P.R. of China

**Phone:** 086-898-88381062

**E-mail:** wangy@idsse.ac.cn

**Supplementary Table 1. Taxonomic abundance of microbial reads in sponges and seawater at the phylum level.** Microbial reads were assigned taxonomically using the RDP classifier against the SILVA 111 database with a confidence threshold of 50%. The abundance was calculated as the proportions of reads assigned to each phylum against all prokaryotic reads in respective sample. Sample IDs are referred to Table 1.

| Taxonomy | SP1-1 | SP1-2 | SP1-3 | SP2-1 | SP2-2 | SP2-3 | SP3-1 | SP3-2 | SP3-3 | SP4-1 | SP4-2 | SP4-3 | SW-1 | SW-2 | SW-3 |
| --- | --- | --- | --- | --- | --- | --- | --- | --- | --- | --- | --- | --- | --- | --- | --- |
| Gammaproteobacteria | 0.1656 | 0.1419 | 0.1453 | 0.1535 | 0.1848 | 0.1498 | 0.1722 | 0.1609 | 0.1739 | 0.1768 | 0.2439 | 0.1817 | 0.1094 | 0.1461 | 0.1223 |
| Alphaproteobacteria | 0.1134 | 0.0874 | 0.1001 | 0.0699 | 0.0750 | 0.0964 | 0.1000 | 0.0967 | 0.0913 | 0.1025 | 0.0903 | 0.0989 | 0.4672 | 0.4217 | 0.4763 |
| Deltaproteobacteria | 0.0461 | 0.0400 | 0.0438 | 0.0581 | 0.0605 | 0.0575 | 0.0434 | 0.0402 | 0.0483 | 0.0525 | 0.0502 | 0.0477 | 0.0028 | 0.0043 | 0.0036 |
| Other Proteobacteria | 0.0091 | 0.0103 | 0.0106 | 0.0246 | 0.0160 | 0.0230 | 0.0126 | 0.0124 | 0.0112 | 0.0169 | 0.0125 | 0.0182 | 0.0012 | 0.0031 | 0.0015 |
| Cyanobacteria | 0.2725 | 0.3590 | 0.2656 | 0.3009 | 0.2486 | 0.1835 | 0.3001 | 0.3359 | 0.2694 | 0.2480 | 0.2592 | 0.2963 | 0.0466 | 0.0546 | 0.0543 |
| Bacteroidetes | 0.0030 | 0.0023 | 0.0021 | 0.0032 | 0.0022 | 0.0024 | 0.0091 | 0.0075 | 0.0043 | 0.0046 | 0.0018 | 0.0044 | 0.3572 | 0.3299 | 0.3209 |
| Chloroflexi | 0.1521 | 0.1421 | 0.1742 | 0.1402 | 0.1707 | 0.1952 | 0.1381 | 0.1274 | 0.1575 | 0.1424 | 0.1307 | 0.1113 | 0.0009 | 0.0049 | 0.0017 |
| Acidobacteria | 0.0984 | 0.1047 | 0.1112 | 0.0933 | 0.0783 | 0.0994 | 0.0860 | 0.0870 | 0.1067 | 0.1029 | 0.0837 | 0.0943 | 0.0003 | 0.0016 | 0.0004 |
| Actinobacteria | 0.0681 | 0.0639 | 0.0763 | 0.0805 | 0.0699 | 0.0686 | 0.0679 | 0.0649 | 0.0702 | 0.0748 | 0.0696 | 0.0831 | 0.0029 | 0.0097 | 0.0047 |
| Thaumarchaeota | 0.0207 | 0.0047 | 0.0151 | 0.0224 | 0.0262 | 0.0490 | 0.0198 | 0.0171 | 0.0161 | 0.0240 | 0.0099 | 0.0142 | 0.0007 | 0.0027 | 0.0011 |
| Gemmatimonadetes | 0.0239 | 0.0270 | 0.0310 | 0.0275 | 0.0219 | 0.0303 | 0.0238 | 0.0302 | 0.0261 | 0.0284 | 0.0277 | 0.0258 | 0.0001 | 0.0004 | 0.0001 |
| Poribacteria | 0.0150 | 0.0096 | 0.0143 | 0.0119 | 0.0227 | 0.0221 | 0.0122 | 0.0095 | 0.0117 | 0.0142 | 0.0115 | 0.0126 | 0.0001 | 0.0007 | 0.0002 |
| Nitrospirae | 0.0059 | 0.0032 | 0.0044 | 0.0062 | 0.0102 | 0.0096 | 0.0076 | 0.0051 | 0.0064 | 0.0052 | 0.0030 | 0.0048 | 0.0004 | 0.0021 | 0.0005 |
| Minor group (<1%) | 0.0060 | 0.0040 | 0.0059 | 0.0078 | 0.0129 | 0.0131 | 0.0071 | 0.0051 | 0.0069 | 0.0069 | 0.0061 | 0.0068 | 0.0102 | 0.0182 | 0.0124 |
|  |  |  |  |  |  |  |  |  |  |  |  |  |  |  |  |
| Minor group | SP1-1 | SP1-2 | SP1-3 | SP2-1 | SP2-2 | SP2-3 | SP3-1 | SP3-2 | SP3-3 | SP4-1 | SP4-2 | SP4-3 | SW-1 | SW-2 | SW-3 |
| Spirochaetes | 0.0029 | 0.0012 | 0.0020 | 0.0034 | 0.0058 | 0.0058 | 0.0033 | 0.0023 | 0.0039 | 0.0029 | 0.0021 | 0.0033 | 0.0001 | 0.0002 | 0.0001 |
| Euryarchaeota | 0.0000 | 0.0000 | 0.0000 | 0.0000 | 0.0000 | 0.0000 | 0.0000 | 0.0000 | 0.0000 | 0.0000 | 0.0000 | 0.0000 | 0.0038 | 0.0053 | 0.0031 |
| Deferribacteres | 0.0022 | 0.0023 | 0.0018 | 0.0029 | 0.0034 | 0.0041 | 0.0031 | 0.0021 | 0.0022 | 0.0033 | 0.0036 | 0.0032 | 0.0006 | 0.0014 | 0.0004 |
| Deinococcus-Thermus | 0.0000 | 0.0000 | 0.0000 | 0.0011 | 0.0032 | 0.0024 | 0.0000 | 0.0000 | 0.0000 | 0.0000 | 0.0000 | 0.0000 | 0.0001 | 0.0002 | 0.0002 |
| Unclassified bacteria | 0.0004 | 0.0004 | 0.0006 | 0.0003 | 0.0003 | 0.0005 | 0.0006 | 0.0005 | 0.0006 | 0.0006 | 0.0003 | 0.0002 | 0.0007 | 0.0031 | 0.0013 |
| Fusobacteria | 0.0000 | 0.0000 | 0.0000 | 0.0000 | 0.0000 | 0.0000 | 0.0000 | 0.0000 | 0.0000 | 0.0000 | 0.0000 | 0.0000 | 0.0012 | 0.0021 | 0.0025 |
| Firmicutes | 0.0001 | 0.0000 | 0.0013 | 0.0001 | 0.0001 | 0.0001 | 0.0001 | 0.0001 | 0.0001 | 0.0000 | 0.0001 | 0.0000 | 0.0013 | 0.0024 | 0.0018 |
| Verrucomicrobia | 0.0003 | 0.0000 | 0.0000 | 0.0000 | 0.0000 | 0.0001 | 0.0000 | 0.0001 | 0.0000 | 0.0000 | 0.0000 | 0.0000 | 0.0017 | 0.0019 | 0.0023 |
| TM7 | 0.0000 | 0.0000 | 0.0000 | 0.0000 | 0.0000 | 0.0000 | 0.0000 | 0.0000 | 0.0000 | 0.0000 | 0.0000 | 0.0000 | 0.0001 | 0.0007 | 0.0002 |
| BD1-5 | 0.0000 | 0.0000 | 0.0000 | 0.0000 | 0.0000 | 0.0000 | 0.0000 | 0.0000 | 0.0000 | 0.0000 | 0.0000 | 0.0000 | 0.0004 | 0.0004 | 0.0003 |
| OD1 | 0.0000 | 0.0000 | 0.0001 | 0.0000 | 0.0001 | 0.0000 | 0.0001 | 0.0000 | 0.0001 | 0.0000 | 0.0000 | 0.0000 | 0.0000 | 0.0001 | 0.0001 |
| Chlorobi | 0.0000 | 0.0000 | 0.0000 | 0.0000 | 0.0000 | 0.0000 | 0.0000 | 0.0000 | 0.0000 | 0.0000 | 0.0000 | 0.0000 | 0.0000 | 0.0001 | 0.0000 |
| Planctomycetes | 0.0000 | 0.0000 | 0.0000 | 0.0000 | 0.0000 | 0.0000 | 0.0000 | 0.0000 | 0.0000 | 0.0000 | 0.0000 | 0.0000 | 0.0000 | 0.0000 | 0.0001 |
| SM2F11 | 0.0000 | 0.0000 | 0.0000 | 0.0000 | 0.0000 | 0.0000 | 0.0000 | 0.0000 | 0.0000 | 0.0000 | 0.0000 | 0.0000 | 0.0000 | 0.0001 | 0.0000 |

**Supplementary Table 2. Highly abundant OTUs in sponges and seawater samples.** Microbial reads were assigned taxonomically using the RDP classifier against the SILVA 111 database with a confidence threshold of 50%. The abundance was calculated as the proportions of reads assigned to each OTU against all prokaryotic reads in respective sample. Sample IDs are referred to Table 1.

| **IDs** | **Taxonomy** | **SP1-1** | **SP1-2** | **SP1-3** | **SP2-1** | **SP2-2** | **SP2-3** | **SP3-1** | **SP3-2** | **SP3-3** | **SP4-1** | **SP4-2** | **SP4-3** | **SW-1** | **SW-2** | **SW-3** |
| --- | --- | --- | --- | --- | --- | --- | --- | --- | --- | --- | --- | --- | --- | --- | --- | --- |
| ***Highly abundant OTUs in sponges*** | | | | | | | | | | | | | | | | |
| OTU_9115 | Cyanobacteria | 0.2662 | 0.3557 | 0.2614 | 0.2978 | 0.2455 | 0.1789 | 0.2963 | 0.3319 | 0.2653 | 0.2449 | 0.2569 | 0.2935 | 0.0001 | 0.0001 | 0.0002 |
| OTU_2092 | Chloroflexi | 0.1051 | 0.1011 | 0.1267 | 0.0811 | 0.1120 | 0.1147 | 0.0862 | 0.0819 | 0.1039 | 0.0749 | 0.0718 | 0.0510 | 0.0001 | 0.0005 | 0.0002 |
| OTU_2093 | Actinobacteria | 0.0524 | 0.0509 | 0.0590 | 0.0586 | 0.0534 | 0.0518 | 0.0511 | 0.0482 | 0.0534 | 0.0495 | 0.0510 | 0.0654 | 0.0001 | 0.0005 | 0.0002 |
| OTU_2094 | Acidobacteria | 0.0570 | 0.0619 | 0.0636 | 0.0497 | 0.0373 | 0.0529 | 0.0473 | 0.0443 | 0.0573 | 0.0614 | 0.0450 | 0.0579 | 0.0001 | 0.0003 | 0.0001 |
| OTU_11426 | Gammaproteobacteria | 0.0321 | 0.0283 | 0.0325 | 0.0326 | 0.0335 | 0.0341 | 0.0361 | 0.0240 | 0.0340 | 0.0456 | 0.0352 | 0.0496 | 0.0001 | 0.0003 | 0.0002 |
| OTU_22626 | Gammaproteobacteria | 0.0285 | 0.0296 | 0.0249 | 0.0329 | 0.0417 | 0.0191 | 0.0239 | 0.0188 | 0.0262 | 0.0225 | 0.0254 | 0.0176 | 0.0001 | 0.0003 | 0.0001 |
| OTU_13685 | Gammaproteobacteria | 0.0223 | 0.0207 | 0.0156 | 0.0220 | 0.0196 | 0.0152 | 0.0402 | 0.0463 | 0.0371 | 0.0264 | 0.0206 | 0.0234 | 0.0000 | 0.0002 | 0.0001 |
| OTU_18091 | Chloroflexi | 0.0129 | 0.0161 | 0.0152 | 0.0211 | 0.0108 | 0.0246 | 0.0206 | 0.0176 | 0.0201 | 0.0348 | 0.0328 | 0.0315 | 0.0001 | 0.0002 | 0.0001 |
| OTU_13688 | Gammaproteobacteria | 0.0150 | 0.0136 | 0.0156 | 0.0252 | 0.0295 | 0.0257 | 0.0150 | 0.0147 | 0.0177 | 0.0145 | 0.0153 | 0.0157 | 0.0000 | 0.0003 | 0.0002 |
| OTU_10953 | Alphaproteobacteria | 0.0331 | 0.0244 | 0.0226 | 0.0017 | 0.0019 | 0.0032 | 0.0251 | 0.0295 | 0.0213 | 0.0161 | 0.0123 | 0.0148 | 0.0000 | 0.0004 | 0.0001 |
| OTU_9111 | Thaumarchaeota | 0.0196 | 0.0033 | 0.0136 | 0.0186 | 0.0162 | 0.0225 | 0.0152 | 0.0131 | 0.0117 | 0.0240 | 0.0099 | 0.0141 | 0.0000 | 0.0000 | 0.0000 |
| OTU_2095 | Deltaproteobacteria | 0.0159 | 0.0103 | 0.0139 | 0.0133 | 0.0172 | 0.0178 | 0.0148 | 0.0126 | 0.0146 | 0.0117 | 0.0120 | 0.0115 | 0.0000 | 0.0001 | 0.0001 |
| OTU_6942 | Alphaproteobacteria | 0.0207 | 0.0138 | 0.0204 | 0.0081 | 0.0124 | 0.0131 | 0.0210 | 0.0184 | 0.0170 | 0.0062 | 0.0062 | 0.0071 | 0.0001 | 0.0002 | 0.0000 |
| OTU_19948 | Alphaproteobacteria | 0.0094 | 0.0083 | 0.0100 | 0.0113 | 0.0147 | 0.0156 | 0.0125 | 0.0105 | 0.0121 | 0.0171 | 0.0114 | 0.0128 | 0.0001 | 0.0006 | 0.0001 |
| OTU_2790 | JTB23 | 0.0068 | 0.0080 | 0.0086 | 0.0115 | 0.0112 | 0.0177 | 0.0093 | 0.0097 | 0.0090 | 0.0140 | 0.0109 | 0.0125 | 0.0001 | 0.0004 | 0.0002 |
| OTU_6641 | Deltaproteobacteria | 0.0084 | 0.0087 | 0.0089 | 0.0079 | 0.0080 | 0.0084 | 0.0077 | 0.0075 | 0.0096 | 0.0181 | 0.0144 | 0.0139 | 0.0000 | 0.0002 | 0.0001 |
| OTU_20816 | Gammaproteobacteria | 0.0003 | 0.0026 | 0.0007 | 0.0005 | 0.0003 | 0.0006 | 0.0007 | 0.0013 | 0.0003 | 0.0093 | 0.0793 | 0.0147 | 0.0000 | 0.0000 | 0.0000 |
| OTU_13506 | Gemmatimonadetes | 0.0127 | 0.0174 | 0.0193 | 0.0000 | 0.0000 | 0.0000 | 0.0114 | 0.0183 | 0.0138 | 0.0034 | 0.0055 | 0.0057 | 0.0000 | 0.0001 | 0.0000 |
| OTU_15341 | Deltaproteobacteria | 0.0084 | 0.0087 | 0.0085 | 0.0066 | 0.0054 | 0.0065 | 0.0073 | 0.0073 | 0.0089 | 0.0117 | 0.0137 | 0.0133 | 0.0000 | 0.0002 | 0.0000 |
| OTU_8116 | Alphaproteobacteria | 0.0052 | 0.0060 | 0.0069 | 0.0089 | 0.0126 | 0.0086 | 0.0051 | 0.0047 | 0.0063 | 0.0139 | 0.0129 | 0.0135 | 0.0000 | 0.0002 | 0.0001 |
| OTU_15340 | Chloroflexi | 0.0077 | 0.0062 | 0.0101 | 0.0070 | 0.0075 | 0.0110 | 0.0083 | 0.0067 | 0.0092 | 0.0119 | 0.0093 | 0.0077 | 0.0001 | 0.0002 | 0.0002 |
| OTU_8295 | Alphaproteobacteria | 0.0095 | 0.0095 | 0.0105 | 0.0077 | 0.0031 | 0.0141 | 0.0069 | 0.0078 | 0.0082 | 0.0059 | 0.0080 | 0.0098 | 0.0000 | 0.0001 | 0.0000 |
| OTU_5156 | Chloroflexi | 0.0068 | 0.0047 | 0.0072 | 0.0083 | 0.0082 | 0.0116 | 0.0089 | 0.0074 | 0.0079 | 0.0094 | 0.0064 | 0.0073 | 0.0000 | 0.0001 | 0.0000 |
| OTU_18235 | Actinobacteria | 0.0059 | 0.0054 | 0.0071 | 0.0117 | 0.0095 | 0.0080 | 0.0068 | 0.0063 | 0.0064 | 0.0109 | 0.0072 | 0.0077 | 0.0000 | 0.0001 | 0.0000 |
| OTU_9113 | Acidobacteria | 0.0054 | 0.0067 | 0.0084 | 0.0013 | 0.0009 | 0.0013 | 0.0051 | 0.0080 | 0.0111 | 0.0143 | 0.0130 | 0.0118 | 0.0000 | 0.0002 | 0.0000 |
| OTU_3616 | Gemmatimonadetes | 0.0000 | 0.0000 | 0.0000 | 0.0178 | 0.0137 | 0.0183 | 0.0003 | 0.0002 | 0.0001 | 0.0139 | 0.0122 | 0.0102 | 0.0000 | 0.0001 | 0.0000 |
| OTU_16455 | Acidobacteria | 0.0068 | 0.0096 | 0.0085 | 0.0068 | 0.0041 | 0.0071 | 0.0076 | 0.0085 | 0.0097 | 0.0057 | 0.0054 | 0.0063 | 0.0000 | 0.0000 | 0.0000 |
| OTU_12671 | Gammaproteobacteria | 0.0060 | 0.0085 | 0.0104 | 0.0057 | 0.0057 | 0.0081 | 0.0057 | 0.0062 | 0.0074 | 0.0077 | 0.0069 | 0.0072 | 0.0000 | 0.0000 | 0.0000 |
| OTU_22145 | Acidobacteria | 0.0062 | 0.0046 | 0.0048 | 0.0119 | 0.0110 | 0.0122 | 0.0035 | 0.0037 | 0.0040 | 0.0060 | 0.0055 | 0.0044 | 0.0001 | 0.0003 | 0.0000 |
| OTU_5132 | Gammaproteobacteria | 0.0003 | 0.0004 | 0.0001 | 0.0020 | 0.0122 | 0.0022 | 0.0034 | 0.0076 | 0.0037 | 0.0078 | 0.0228 | 0.0140 | 0.0000 | 0.0000 | 0.0000 |
| OTU_14043 | Acidobacteria | 0.0068 | 0.0066 | 0.0067 | 0.0067 | 0.0058 | 0.0047 | 0.0078 | 0.0065 | 0.0073 | 0.0059 | 0.0044 | 0.0053 | 0.0000 | 0.0001 | 0.0000 |
| OTU_4807 | Nitrospirae | 0.0059 | 0.0032 | 0.0044 | 0.0062 | 0.0101 | 0.0096 | 0.0076 | 0.0051 | 0.0064 | 0.0052 | 0.0030 | 0.0048 | 0.0004 | 0.0018 | 0.0004 |
| OTU_20952 | Gammaproteobacteria | 0.0113 | 0.0039 | 0.0055 | 0.0056 | 0.0059 | 0.0081 | 0.0071 | 0.0050 | 0.0055 | 0.0038 | 0.0030 | 0.0051 | 0.0000 | 0.0000 | 0.0000 |
| OTU_9116 | Alphaproteobacteria | 0.0063 | 0.0041 | 0.0065 | 0.0041 | 0.0052 | 0.0055 | 0.0075 | 0.0069 | 0.0055 | 0.0058 | 0.0066 | 0.0052 | 0.0000 | 0.0002 | 0.0001 |
| OTU_10957 | Gammaproteobacteria | 0.0051 | 0.0057 | 0.0068 | 0.0051 | 0.0041 | 0.0079 | 0.0038 | 0.0034 | 0.0037 | 0.0072 | 0.0063 | 0.0079 | 0.0000 | 0.0001 | 0.0000 |
| OTU_4810 | Gammaproteobacteria | 0.0063 | 0.0034 | 0.0039 | 0.0040 | 0.0051 | 0.0059 | 0.0070 | 0.0076 | 0.0066 | 0.0047 | 0.0059 | 0.0051 | 0.0000 | 0.0000 | 0.0000 |
| OTU_18894 | Chloroflexi | 0.0062 | 0.0043 | 0.0047 | 0.0051 | 0.0093 | 0.0115 | 0.0051 | 0.0045 | 0.0060 | 0.0017 | 0.0036 | 0.0027 | 0.0000 | 0.0003 | 0.0001 |
| OTU_4609 | Actinobacteria | 0.0045 | 0.0045 | 0.0049 | 0.0046 | 0.0031 | 0.0040 | 0.0058 | 0.0056 | 0.0057 | 0.0071 | 0.0069 | 0.0066 | 0.0000 | 0.0002 | 0.0000 |
| OTU_16354 | Alphaproteobacteria | 0.0025 | 0.0047 | 0.0036 | 0.0072 | 0.0041 | 0.0102 | 0.0025 | 0.0021 | 0.0025 | 0.0045 | 0.0098 | 0.0077 | 0.0000 | 0.0001 | 0.0000 |
| OTU_18569 | Poribacteria | 0.0099 | 0.0061 | 0.0093 | 0.0015 | 0.0032 | 0.0035 | 0.0072 | 0.0054 | 0.0070 | 0.0026 | 0.0020 | 0.0035 | 0.0000 | 0.0002 | 0.0000 |
| ***Highly abundant OTUs in sponges*** | | | | | | | | | | | | | | | | |
| OTU_12359 | Bacteroidetes | 0.0000 | 0.0000 | 0.0000 | 0.0001 | 0.0001 | 0.0000 | 0.0000 | 0.0000 | 0.0000 | 0.0000 | 0.0000 | 0.0000 | 0.2802 | 0.2372 | 0.2455 |
| OTU_19042 | Alphaproteobacteria | 0.0000 | 0.0000 | 0.0000 | 0.0000 | 0.0000 | 0.0000 | 0.0000 | 0.0000 | 0.0000 | 0.0000 | 0.0000 | 0.0000 | 0.1280 | 0.1033 | 0.1040 |
| OTU_13955 | Alphaproteobacteria | 0.0000 | 0.0000 | 0.0000 | 0.0000 | 0.0000 | 0.0000 | 0.0000 | 0.0000 | 0.0000 | 0.0000 | 0.0000 | 0.0000 | 0.1050 | 0.0818 | 0.1076 |
| OTU_19113 | Cyanobacteria | 0.0020 | 0.0018 | 0.0022 | 0.0017 | 0.0022 | 0.0036 | 0.0019 | 0.0010 | 0.0018 | 0.0016 | 0.0015 | 0.0017 | 0.0409 | 0.0436 | 0.0475 |
| OTU_19500 | Gammaproteobacteria | 0.0000 | 0.0000 | 0.0000 | 0.0000 | 0.0000 | 0.0000 | 0.0000 | 0.0000 | 0.0000 | 0.0000 | 0.0000 | 0.0000 | 0.0432 | 0.0348 | 0.0340 |
| OTU_11874 | Alphaproteobacteria | 0.0000 | 0.0000 | 0.0000 | 0.0000 | 0.0000 | 0.0000 | 0.0000 | 0.0000 | 0.0000 | 0.0000 | 0.0000 | 0.0000 | 0.0299 | 0.0257 | 0.0403 |
| OTU_11522 | Bacteroidetes | 0.0000 | 0.0000 | 0.0000 | 0.0000 | 0.0000 | 0.0000 | 0.0001 | 0.0000 | 0.0000 | 0.0000 | 0.0000 | 0.0000 | 0.0310 | 0.0342 | 0.0282 |
| OTU_14492 | Alphaproteobacteria | 0.0000 | 0.0001 | 0.0000 | 0.0000 | 0.0000 | 0.0000 | 0.0000 | 0.0000 | 0.0000 | 0.0000 | 0.0000 | 0.0000 | 0.0209 | 0.0136 | 0.0346 |
| OTU_17140 | Alphaproteobacteria | 0.0000 | 0.0000 | 0.0000 | 0.0000 | 0.0000 | 0.0000 | 0.0000 | 0.0000 | 0.0000 | 0.0000 | 0.0000 | 0.0000 | 0.0226 | 0.0189 | 0.0174 |
| OTU_14056 | Alphaproteobacteria | 0.0000 | 0.0000 | 0.0000 | 0.0000 | 0.0000 | 0.0000 | 0.0000 | 0.0000 | 0.0000 | 0.0000 | 0.0000 | 0.0000 | 0.0234 | 0.0198 | 0.0145 |
| OTU_15636 | Bacteroidetes | 0.0000 | 0.0000 | 0.0000 | 0.0000 | 0.0000 | 0.0001 | 0.0000 | 0.0000 | 0.0000 | 0.0000 | 0.0000 | 0.0000 | 0.0198 | 0.0188 | 0.0178 |
| OTU_18990 | Alphaproteobacteria | 0.0000 | 0.0000 | 0.0000 | 0.0000 | 0.0000 | 0.0000 | 0.0000 | 0.0000 | 0.0000 | 0.0000 | 0.0000 | 0.0000 | 0.0198 | 0.0171 | 0.0151 |
| OTU_5575 | Alphaproteobacteria | 0.0002 | 0.0000 | 0.0000 | 0.0000 | 0.0000 | 0.0000 | 0.0000 | 0.0000 | 0.0000 | 0.0000 | 0.0000 | 0.0000 | 0.0187 | 0.0154 | 0.0169 |
| OTU_15421 | Gammaproteobacteria | 0.0000 | 0.0000 | 0.0000 | 0.0000 | 0.0000 | 0.0000 | 0.0000 | 0.0000 | 0.0000 | 0.0000 | 0.0000 | 0.0000 | 0.0030 | 0.0315 | 0.0075 |
| OTU_20670 | Alphaproteobacteria | 0.0000 | 0.0000 | 0.0000 | 0.0000 | 0.0001 | 0.0000 | 0.0001 | 0.0000 | 0.0001 | 0.0000 | 0.0000 | 0.0000 | 0.0143 | 0.0161 | 0.0102 |
| OTU_10498 | Gammaproteobacteria | 0.0000 | 0.0000 | 0.0000 | 0.0000 | 0.0000 | 0.0000 | 0.0000 | 0.0000 | 0.0000 | 0.0000 | 0.0000 | 0.0000 | 0.0147 | 0.0130 | 0.0112 |
| OTU_11526 | Alphaproteobacteria | 0.0000 | 0.0000 | 0.0000 | 0.0000 | 0.0000 | 0.0000 | 0.0000 | 0.0000 | 0.0000 | 0.0000 | 0.0000 | 0.0000 | 0.0092 | 0.0083 | 0.0070 |
| OTU_9735 | Gammaproteobacteria | 0.0002 | 0.0002 | 0.0001 | 0.0000 | 0.0000 | 0.0001 | 0.0001 | 0.0000 | 0.0001 | 0.0000 | 0.0001 | 0.0000 | 0.0071 | 0.0061 | 0.0113 |
| OTU_16643 | Alphaproteobacteria | 0.0000 | 0.0000 | 0.0000 | 0.0000 | 0.0000 | 0.0000 | 0.0000 | 0.0000 | 0.0000 | 0.0000 | 0.0000 | 0.0000 | 0.0050 | 0.0073 | 0.0095 |
| OTU_13424 | Alphaproteobacteria | 0.0000 | 0.0000 | 0.0000 | 0.0000 | 0.0000 | 0.0000 | 0.0000 | 0.0000 | 0.0000 | 0.0000 | 0.0000 | 0.0000 | 0.0058 | 0.0040 | 0.0090 |
| OTU_892 | Alphaproteobacteria | 0.0000 | 0.0000 | 0.0000 | 0.0000 | 0.0000 | 0.0000 | 0.0000 | 0.0000 | 0.0000 | 0.0000 | 0.0000 | 0.0000 | 0.0048 | 0.0061 | 0.0057 |

**Supplementary Table 3. Number of Eukaryotic-like domains in Chloroflexi symbiont and it closed relatives.** Predicted amino acids of each genome was annotated using pfam_scan.pl script by searching against the PFAM database. Eukaryotic-like domains were summarized using Pfam IDs. *Taxa*: 1, Chloroflexi Sp. DE2092 in the present study; 2, *Anaerolinea thermolimosa* (Genome accession number: BBXW01000001); 3, *Anaerolinea thermophila* (NC_014960); 4, *Ardenticatena maritima* (LGKN01000001); 5, *Bellilinea caldifistulae* (LGHJ01000001); 6, *Caldilinea aerophila* (NC_017079); 7, *Dehalogenimonas alkenigignens* (LFDV01000001); 8, *Dehalogenimonas lykanthroporepellens* (NC_014314); 9, *Dehalococcoides mccartyi* (NC_002936); 10, Levilinea saccharolytica (BBXZ01000001); 11, *Leptolinea tardivitalis* (BBYA01000001); 12, *Longilinea arvoryzae* (BBXY01000001); 13, *Ornatilinea apprima* (LGCL01000001); 14, *Thermoflexus hugenholtzii* (NA).

|  | Taxa | | | | | | | | | | | | | |
| --- | --- | --- | --- | --- | --- | --- | --- | --- | --- | --- | --- | --- | --- | --- |
|  | **1** | **2** | **3** | **4** | **5** | **6** | **7** | **8** | **9** | **10** | **11** | **12** | **13** | **14** |
| Eukaryotic-like domains |  |  |  |  |  |  |  |  |  |  |  |  |  |  |
| Fibronectin type III domain | 34 | 5 | 5 | 6 | 1 | 6 | 2 | 0 | 1 | 7 | 1 | 5 | 5 | 1 |
| Cadherin | 0 | 0 | 0 | 0 | 0 | 0 | 0 | 0 | 0 | 5 | 0 | 2 | 2 | 0 |
| Ankyrin repeat | 2 | 0 | 0 | 0 | 0 | 0 | 0 | 0 | 0 | 0 | 0 | 0 | 0 | 0 |
| Tetratricopeptide repeat | 25 | 44 | 39 | 64 | 49 | 50 | 3 | 1 | 0 | 25 | 32 | 55 | 54 | 23 |
| Leucine Rich Repeat | 0 | 1 | 2 | 0 | 2 | 0 | 0 | 0 | 0 | 10 | 1 | 12 | 9 | 0 |
| NHL repeat | 1 | 5 | 5 | 4 | 6 | 12 | 0 | 0 | 2 | 20 | 5 | 4 | 4 | 0 |


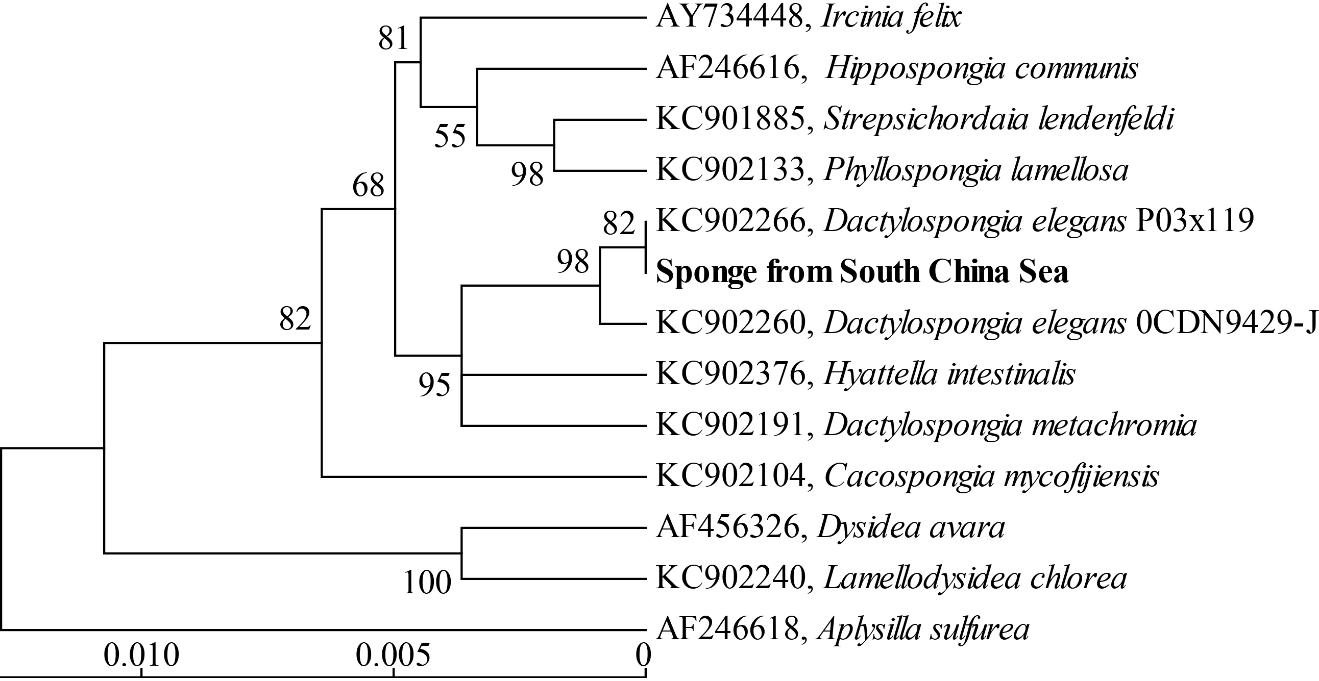


**Supplementary Figure 1. Phylogenetic tree of the 18S rRNA genes of the sponge and its relatives.** The tree was constructed using the neighbor-joining method. Bootstrap values are expressed based on 1000 replications. Bar, 1% estimated sequences divergence.


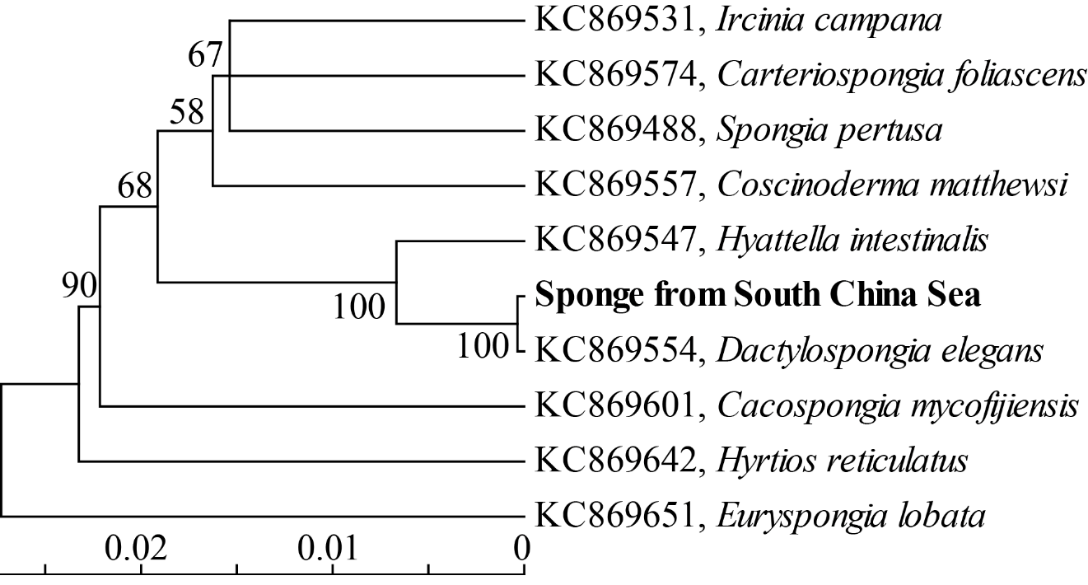


**Supplementary Figure 2. Phylogenetic tree of the 28S rRNA genes of the sponge and its relatives.** The tree was constructed based using the neighbor-joining method. Bootstrap values are expressed based on 1000 replications. Bar, 2% estimated sequences divergence.


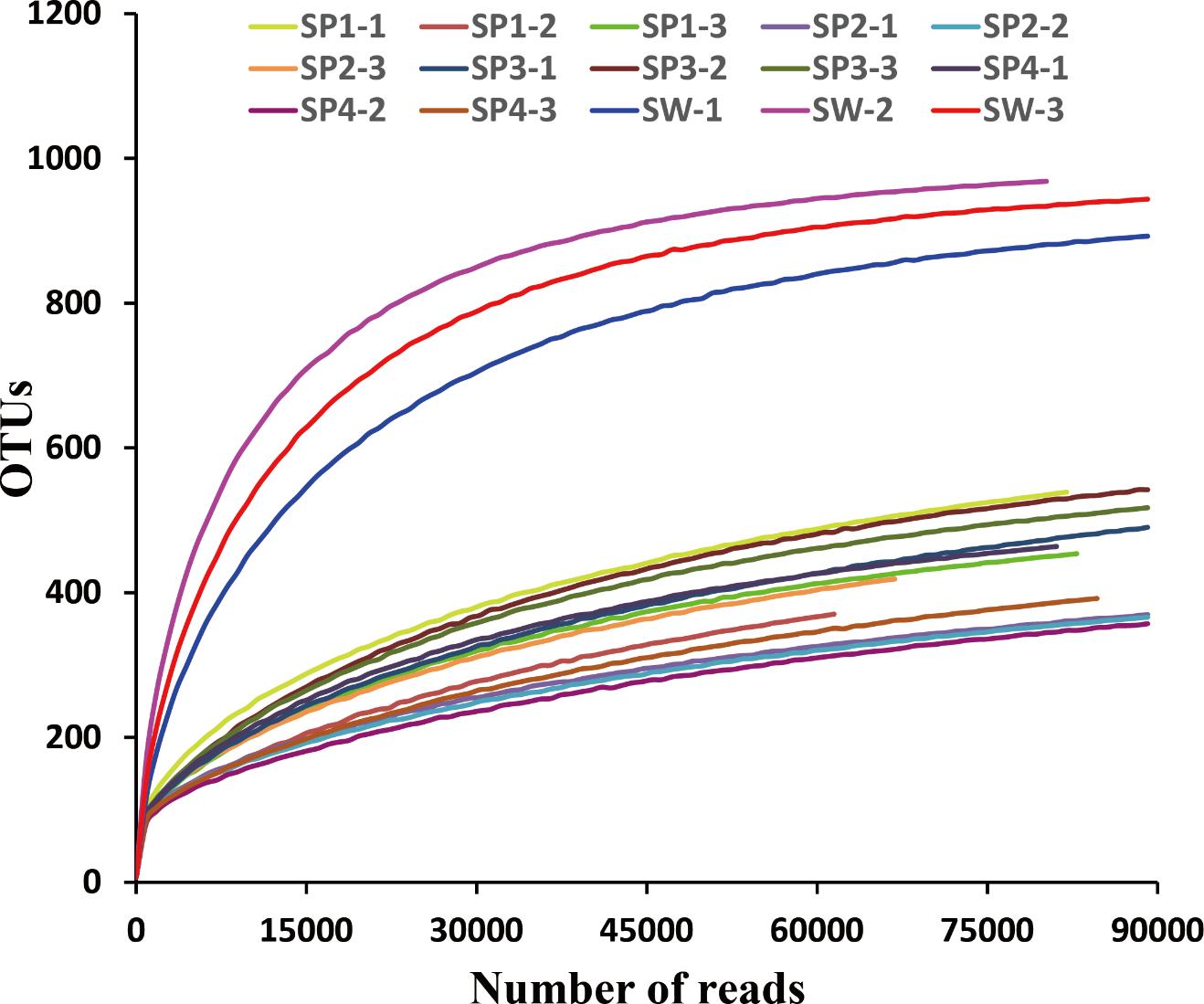


**Supplementary Figure 3. Rarefaction curves showed the microbial diversity in sponges and seawater.** The curves were drawn based on the number of OTUs at a 3% dissimilarity level. Sample IDs are referred to Table 1.

**
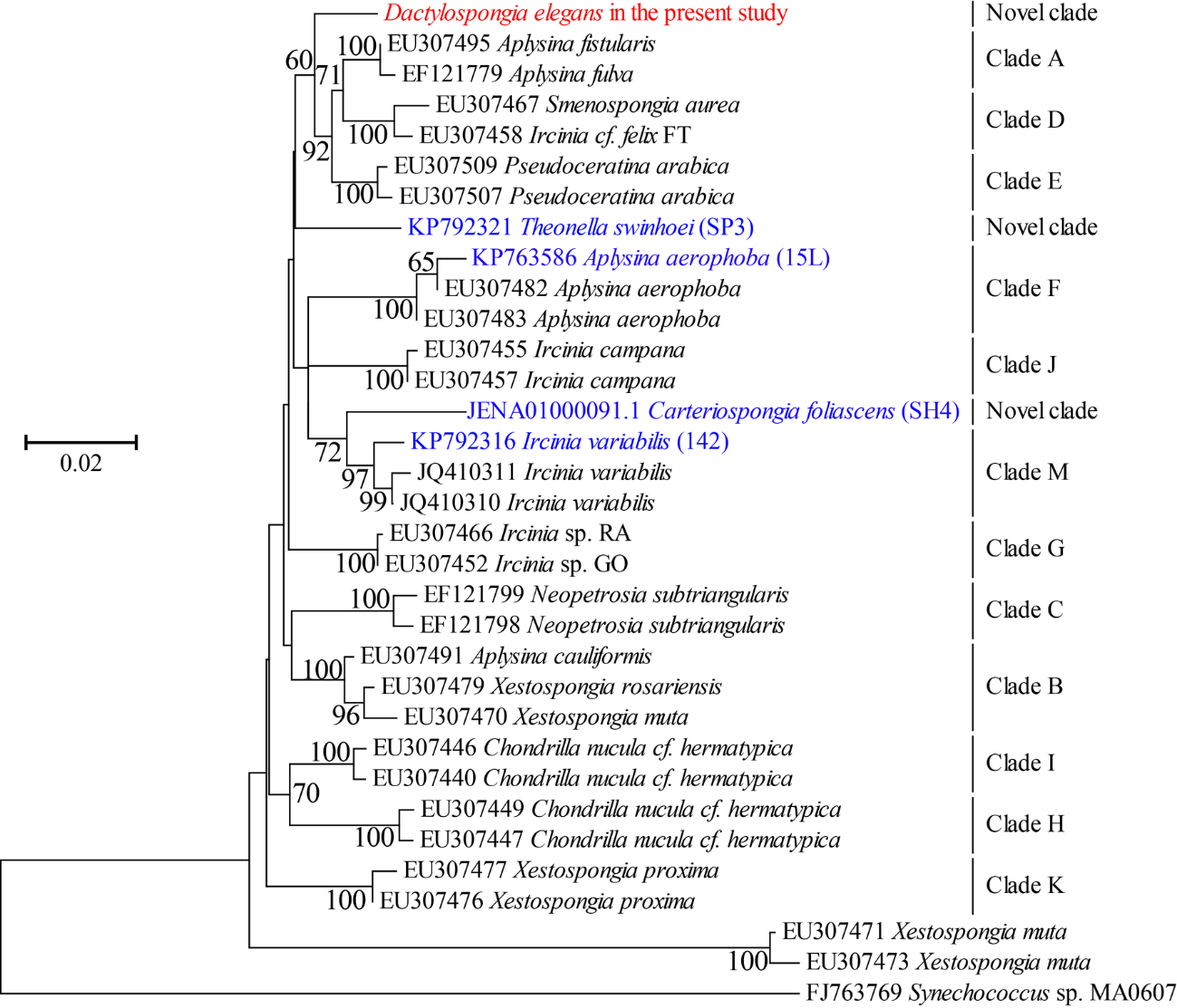
**

**Supplementary Figure 4. Phylogenetic tree of the sponge symbiont “*Ca*. Synechococcus spongiarum”.** The tree was constructed based on the 16S-23S ITS region and partial 16S rRNA gene sequences by the neighbor-joining method. Names on the tree are those of the host sponge species. The previous reported symbionts with genomes were labeled with blue color, and that in the present study were labeled with red color. Bootstrap values are expressed based on 1000 replications, and only values more than 50% are shown. Bar, 2% estimated sequences divergence.

**
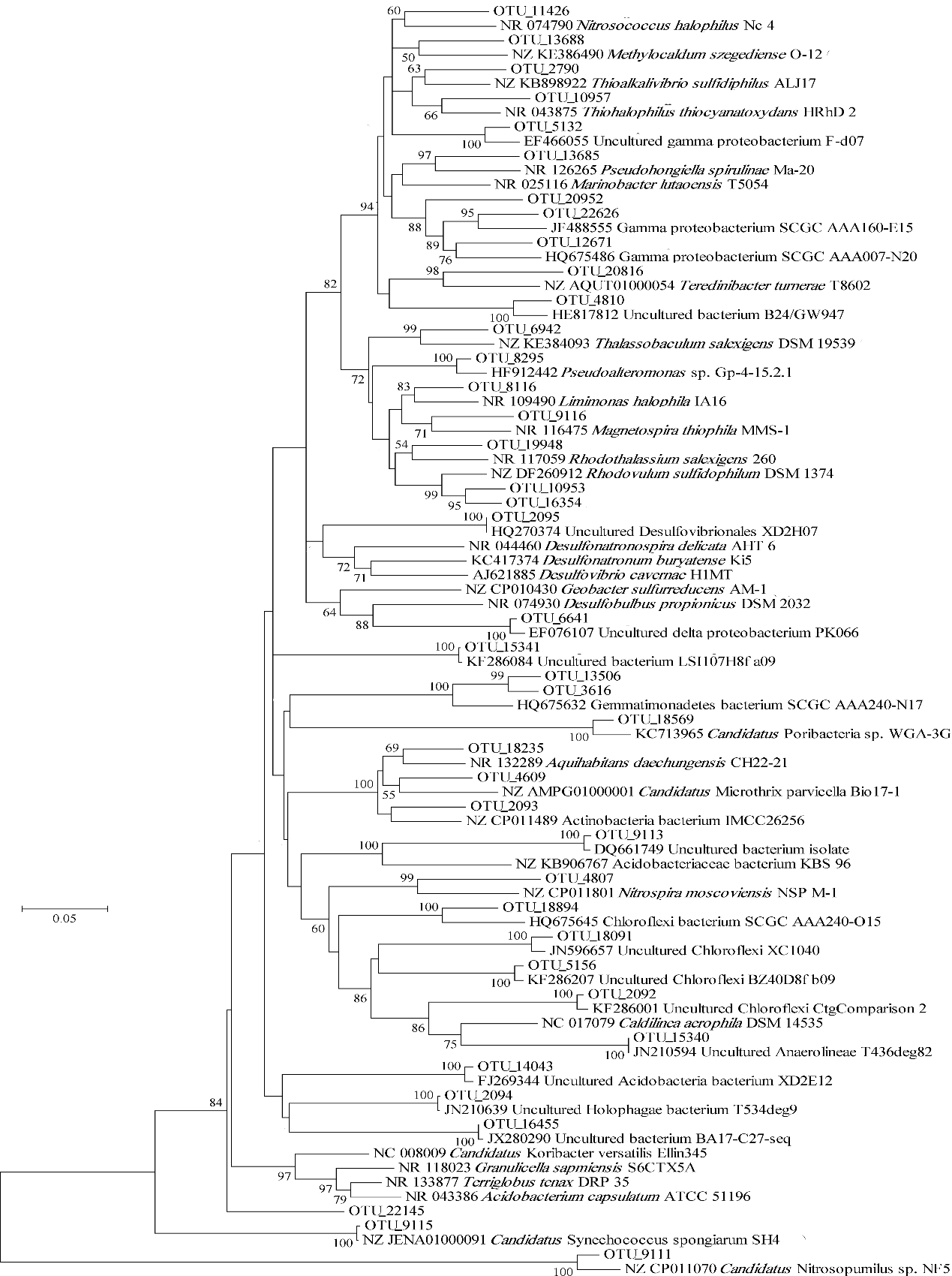
**

**Supplementary Figure 5. Phylogenetic tree of full dataset of the highly abundant OTUs and their relatives.** The tree was constructed based on partial 16S rRNA gene sequences (~420 bp) by the neighbor-joining method. Bootstrap values are expressed based on 1000 replications, and only values more than 50% are shown. Bar, 5% estimated sequences divergence. Relative abundance of OTUs on the tree were shown in Fig. 3.

**
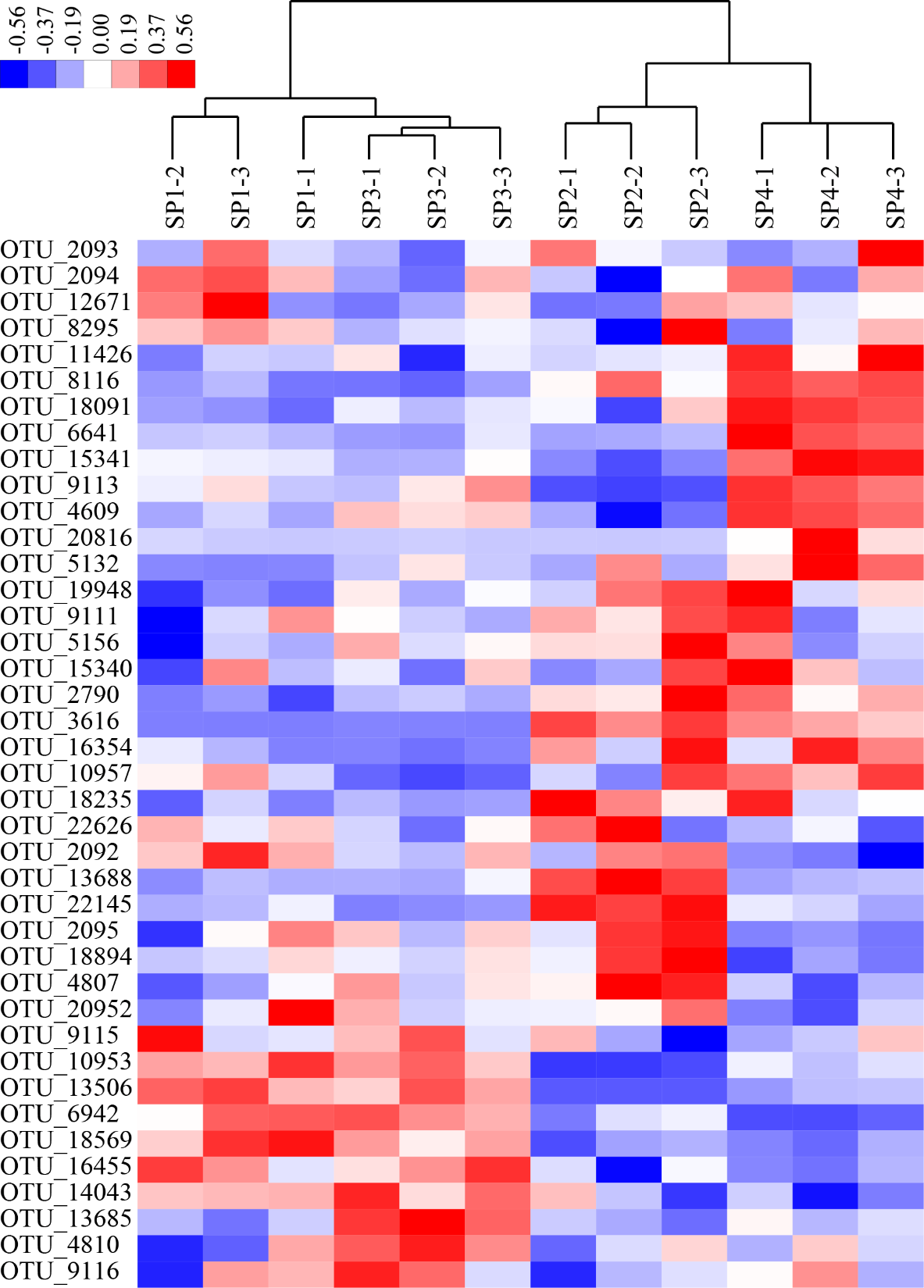
**

**Supplementary Figure 6. Heatmap showing the relative abundance of sponge symbiotic OTUs.** OTUs with relative abundance of less than 0.5% among all the samples were filtered out. The remaining was normalized, centered by the mean, and clustered using the complete linkage method and a metric of correlation (uncentered). The color code indicates differences in relative abundance from the mean, ranging from *blue* (negative) through *white* (the mean) to *red* (positive). Sample IDs are referred to Table 1.


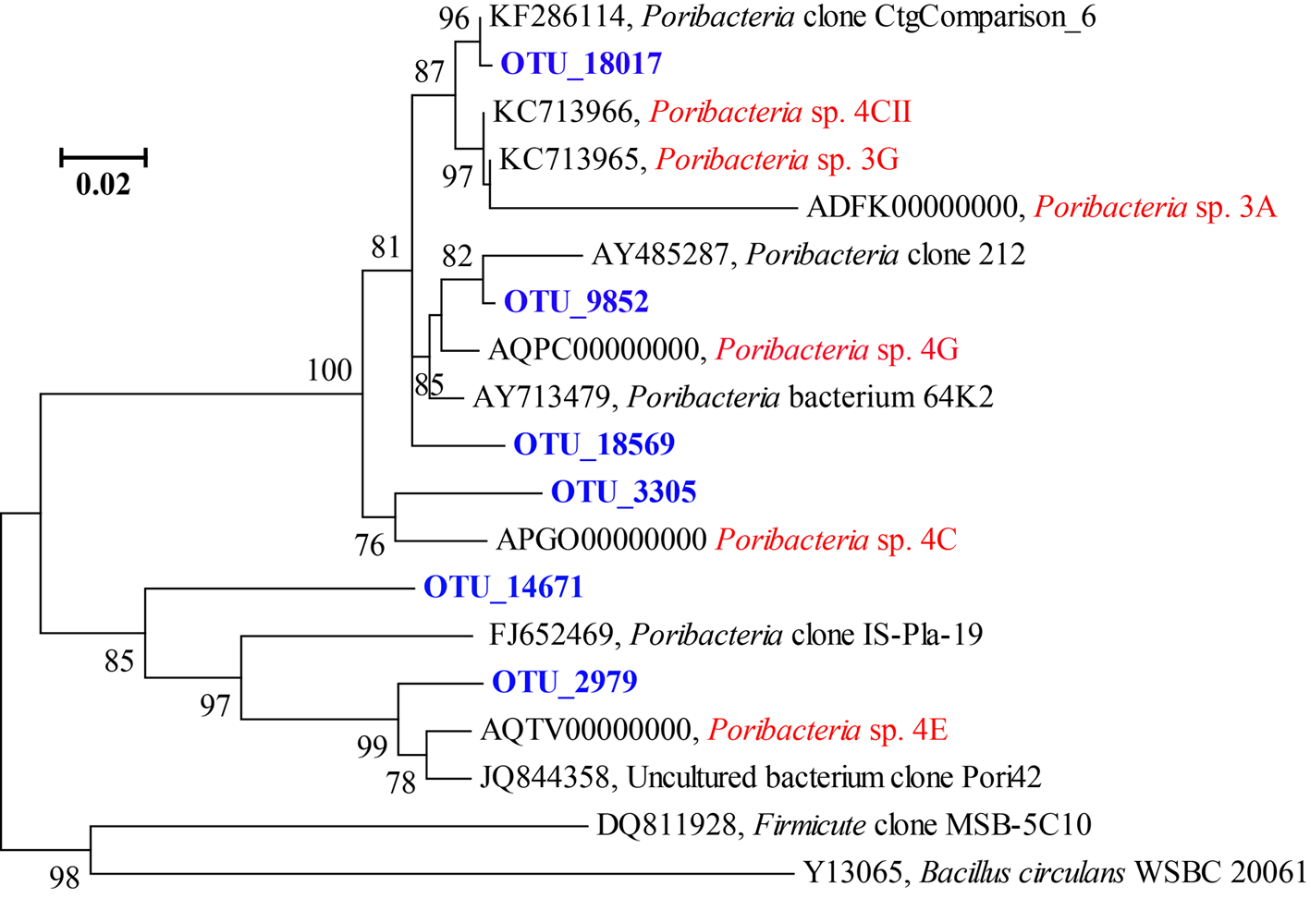


**Supplementary Figure 7. Phylogenetic tree of poribacterial OTUs and their relatives.** The tree was constructed based on partial 16S rRNA gene sequences (424 bp) by the neighbor-joining method. Bootstrap values are expressed based on 1000 replications, and only values more than 50% are shown. Bar, 2% estimated sequences divergence. OTUs from the present work were labeled with blue color. Previous poribacterial symbionts with reported genomes were labeled with red color.
